# The Terrestrial Carnivorous Plant *Utricularia reniformis* Sheds Light on Environmental and Life-Form Genome Plasticity

**DOI:** 10.3390/ijms21010003

**Published:** 2019-12-18

**Authors:** Saura R. Silva, Ana Paula Moraes, Helen A. Penha, Maria H. M. Julião, Douglas S. Domingues, Todd P. Michael, Vitor F. O. Miranda, Alessandro M. Varani

**Affiliations:** 1 Departamento de Tecnologia, Faculdade de Ciências Agrárias e Veterinárias, UNESP—Universidade Estadual Paulista, Jaboticabal 14884-900, Brazil; saura.silva@gmail.com (S.R.S.); helen.penha@gmail.com (H.A.P.); mhmjuliao@gmail.com (M.H.M.J.); 2 Centro de Ciências Naturais e Humanas, Universidade Federal do ABC, São Bernardo do Campo 09606-070, Brazil; apaulademoraes@gmail.com; 3 Departamento de Botânica, Instituto de Biociências, UNESP—Universidade Estadual Paulista, Rio Claro 13506-900, Brazil; douglas.domingues@unesp.br; 4 J. Craig Venter Institute, La Jolla, CA 92037, USA; tmichael@jcvi.org; 5 Departamento de Biologia Aplicada à Agropecuária, Faculdade de Ciências Agrárias e Veterinárias, UNESP—Universidade Estadual Paulista, Jaboticabal 14884-900, Brazil

**Keywords:** evolution, genome fractionation, ABC transporters, transcription factors, transposable elements, whole-genome duplication

## Abstract

*Utricularia* belongs to Lentibulariaceae, a widespread family of carnivorous plants that possess ultra-small and highly dynamic nuclear genomes. It has been shown that the Lentibulariaceae genomes have been shaped by transposable elements expansion and loss, and multiple rounds of whole-genome duplications (WGD), making the family a platform for evolutionary and comparative genomics studies. To explore the evolution of *Utricularia*, we estimated the chromosome number and genome size, as well as sequenced the terrestrial bladderwort *Utricularia reniformis* (2*n* = 40, 1C = 317.1-Mpb). Here, we report a high quality 304 Mb draft genome, with a scaffold NG50 of 466-Kb, a BUSCO completeness of 87.8%, and 42,582 predicted genes. Compared to the smaller and aquatic *U. gibba* genome (101 Mb) that has a 32% repetitive sequence, the *U. reniformis* genome is highly repetitive (56%). The structural differences between the two genomes are the result of distinct fractionation and rearrangements after WGD, and massive proliferation of LTR-retrotransposons. Moreover, GO enrichment analyses suggest an ongoing gene birth–death–innovation process occurring among the tandem duplicated genes, shaping the evolution of carnivory-associated functions. We also identified unique patterns of developmentally related genes that support the terrestrial life-form and body plan of *U. reniformis*. Collectively, our results provided additional insights into the evolution of the plastic and specialized Lentibulariaceae genomes.

## 1. Introduction

The carnivorous plants from the Lentibulariaceae Rich. family exhibit different life-forms according to their habitats (terrestrial, aquatic, lithophytes, epiphytes, and rheophytes), showing an enormous diversity and worldwide distribution [1]. The family is composed of three genera: *Genlisea* A. St.-Hil. (corkscrew plants), *Pinguicula* L. (butterworts), and *Utricularia* L. (bladderworts). The sequencing of four Lentibulariaceae genomes: *Genlisea aurea* A. St.-Hil. [2], *G. nigrocaulis* Steyerm, *G. hispidula* Stapf [3], and *Utricularia gibba* L. [4,5], along with the estimation of genome size [6,7,8] and karyological analyses (e.g., [3,9,10,11,12]), revealed a wide distribution of genome size and chromosome numbers. In *Utricularia*, genome size ranges from ultra-small (~79 Mb) in *U. purpurea* Walter to large (~706 Mb) in *U. caerulea* L., with a variable number of chromosomes (e.g., 2*n* = 18, 28, 30, 36, 38, 40, 44, 48, and 56), supporting the occurrence of polyploidy and aneuploidy/dysploidy (for review see [8,12]).

Lentibulariaceae genome shrinkage has been attributed to a decrease in the number and length of introns and intergenic regions, associated with transposable elements (TEs) silencing. This phenomenon was primarily observed in *U. gibba* (101 Mb) and *G. aurea* (63–131 Mb according to [8,12]) genomes [2,13,14]. One of the leading hypotheses to explain the genome shrinkage is based on the high respiration rates caused by the carnivorous traps [15], which can increase reactive oxygen species (ROS) formation. Moreover, the *U. gibba* and the 86 Mb *G. nigrocaulis* genomes have suffered high rates of gene deletion in comparison to other eudicots, and in the former, a positive selection of tandemly repeated genes implicated in the adaptation to the carnivorous habit is observed [3,5,16].

In contrast, genome size expansion is generally attributed to TEs proliferation, whole-genome duplication (WGD) (represented by retained polyploid duplicates), and small-scale duplication (mainly represented by proximal and tandem copies) [17,18], which are the standard processes for plant genome evolution [19,20]. Together, TEs and WGDs are considered to be the key players in generating genomic novelties and genomic diversification [21,22,23]. Even among the minimal Lentibulariaceae genomes, the importance of these events it is clear, since the changes in size between the large, 1.5-Gb genome of *G. hispidula* and that of *G. nigrocaulis* (86 Mb) are consequences of WGDs, along with the LTR-retrotransposons expansion in the former, and the double-strand break repair-mediated deletions in the latter [3]. Additionally, the LTR-retrotransposons silencing and significant fractionation, which returned several genes to a single copy after multiple rounds of WGDs molded the *U. gibba* genome [4]. Therefore, the dynamic and specialized Lentibulariaceae genomes can provide a natural platform for the study of genome duplication, fractionation, and TEs expansion and silencing, and their impact on the evolution of the angiosperms [4,5,8].

*Utricularia reniformis* A.St.-Hil. is endemic to the Brazilian Atlantic Forest, growing as a terrestrial species in wet grasslands or as an epiphyte in moist habitats [1,24]. Some but limited information is available on the *U. reniformis* nuclear genome, such as its high levels of polymorphism [25], its genome size of 292 Mb (which is an intermediate size in comparison to other *Utricularia*) and a GC content of 38% [8]. We have also previously reported the sequence analyses of *U. reniformis* chloroplast (cpDNA) and mitochondrial (mtDNA) genomes [26,27]. In order to develop insights into the forces that have shaped the genomes of the Lentibulariaceae species, here we estimated the genome size and chromosome number, and deep-sequenced the terrestrial bladderwort *U. reniformis* genome and transcriptome. We also compared the *U. reniformis* draft genome against the aquatic species *U. gibba* and other angiosperms, revealing that the *U. reniformis* has a distinct genome structure, mostly due to lineage-specific WGDs and a TE expansion, reflecting a diversified repertoire of plant developmental and carnivory-associated genes and their underlying terrestrial adaptation.

## 2. Results

### 2.1. The Utricularia reniformis Genome

**Figure 1:**
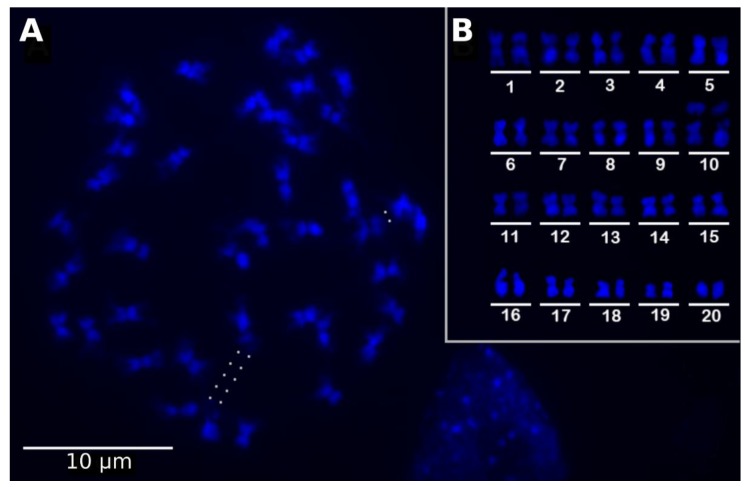
*Utricularia reniformis* metaphases with 2*n* = 40. (**A**) Metaphase stained with DAPI. (**B**) Karyogram using the same metaphase presented in (**A**). Dots in (**A**) represent the distended and unstained region. The bar is equivalent to 10 µm.

We first determined the chromosome number of *U. reniformis* as 2*n* = 40 (Figure 1A,B). The small chromosomes (~1.65 µm) varied between metacentric to submetacentric, with one pair holding a distended satellite, probably representing the ribosomal DNA 45S sites (see dots in Figure 1A and the chromosome pair 10 in Figure 1B). The genome size, estimated by the flow cytometry of the nuclei from leaves, was found to be 2C = 0.652 pg (SD = 1.48%), which represents a haploid genome size of 1C = 317.1 Mb (or 0.324 pg) (Table S1).

**Figure 2:**
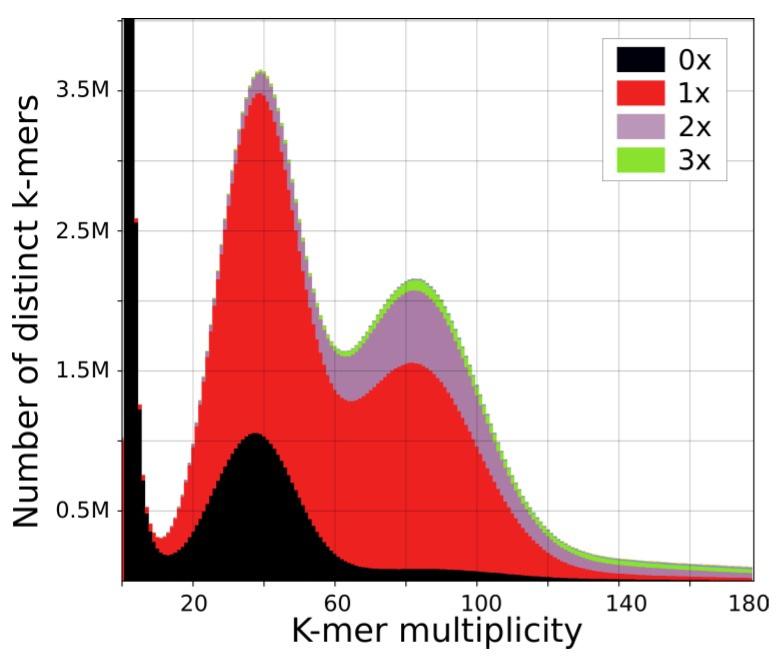
Genome assembly analysis: Read k-mer frequency versus *Utricularia reniformis* assembly copy number stacked histograms generated by the KAT comp tool. Read content in black is absent from the assembly, red occurs once, purple twice, and green occurs three times, indicating a scenario of both heterozygous and polyploidy genome. The heterozygous content is represented by the first peak at x = 40 and the homozygous content in the second peak at x = 80. The hidden content (the black peak) represents the heterozygous content that is lost when the assembly bubbles are collapsed. The genome assembly contains most (but not all) of the heterozygous content and introduces duplications on both heterozygous and homozygous content.

We generated 350 gigabytes of genomic and transcriptomic data for *U. reniformis* (Table 1). The k-mer frequency analysis demonstrated that *U. reniformis* exhibits a high heterozygosity rate of 1.8% (Figure 2 and Figure S1). In addition, the k-mer frequency plot was consistent with a tetraploid genome with the haploid at 149 Mb. Moreover, the predicted genome unique content and repeated content lengths are ~88 Mb (29%) and ~220 Mb (71%), respectively.

The assembled draft genome spanned 304 Mb (96% of the flow cytometry-estimated genome size), with an average coverage of 145× (based on the number and length of aligned reads, divided by the assembled genome size), had an NG50 of 466-Kb and a BUSCO completeness of 87.8%, showing a duplication rate of ~26%, which might correspond with a partial genome duplication (Table S2) and 42,582 gene model predictions. The estimated number of error-free bases was 93% (283 of 304 Mb), which also indicated that multiple repeated genomic regions (e.g., non-autonomous LTR elements, centromeres, and telomeres) remained partial or non-assembled. However, this finding also supported that a large amount of the heterozygous content present in *U. reniformis* genome was represented in the assembled draft genome (Table 2 and Figure 2).

Moreover, several cpDNA- and mtDNA-derived regions were also identified, thus, providing support for the lateral transfer between the organelles and the nuclear genome, as previously reported [26,27]. We also detected simple sequence repeat (SSRs) markers already developed for *U. reniformis* [25], in our draft genome. Among all markers, for two SSR loci, we found corresponding regions in *U. gibba* and *U. reniformis* (Table S3). In both cases, we recovered two scaffolds in *U. reniformis* and only one syntenic genomic region in *U. gibba* (Figure S2). For all other markers, we were not able to detect loci shared in both genomes. In addition to the *Arabidopsis*-type telomeric repeats identified at the scaffolds ends, we also found *Chlamydomonas*- and *Genlisea*-type telomeric repeats in their vicinity, as previously observed for *U. gibba* [5]. Furthermore, *U. reniformis* exhibited a considerable amount of TE-derived sequences, when compared to *U. gibba* (56% vs. 32%).

### 2.2. Structural Comparative Analysis

In order to infer *U. reniformis* genome structure and WGD history, we compared the draft genome to *U. gibba* and other angiosperms. In spite of the low assembly contiguity, we were able to detect at least one WGD round since the core eudicot γ whole-genome triplication (WGT), according to the Ks values for estimates of individual genome duplication events (Figure S3A). Moreover, blockwise relationships between *U. reniformis* and *U. gibba* were about 4:2 (Figure S3B), supporting that *U. reniformis* is a tetraploid, as compared to *U. gibba*. This finding also suggested that the two *Utricularia* genomes responded distinctly after the WGD events.

**Figure 3:**
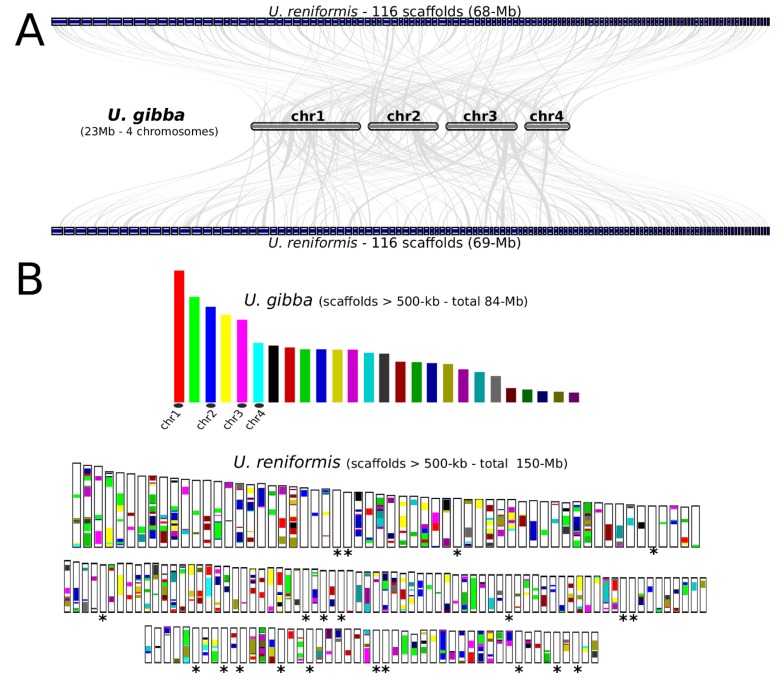
*Utricularia reniformis* vs. *U. gibba* genome comparisons. (**A**) Macrosynteny karyotype visualization of *U. gibba* chromosome 1 (CM007989.1), 2 (CM007990.1), 3 (CM007991.1), and 4 (CM007992.1), against the corresponding *U. reniformis* scaffolds showing matches (generated with the MCscan tool with minspan = 10 option). (**B**) Dual synteny bar plot, created by the ‘*dual-bar plotter*’ from the MCScanX. The upper panel represents *U. gibba* chromosomes and scaffolds; the lower panel represents the *U. reniformis* scaffolds. The syntenic blocks are shown in colors. Most of the white blocks present in each *U. reniformis* scaffolds corresponds to exclusive regions and transposable elements. The asterisks correspond to the *U. reniformis* scaffolds showing no matches to *U. gibba* chromosomes and scaffolds.

The *U. reniformis* and *U. gibba* structural genome differences become more obvious by comparative pairwise alignment. We observed 77% (SD:4%) average nucleotide identity (ANI) between the *U. reniformis* and *U. gibba* assemblies by reciprocal best blast (RBB). Additionally, we observed low global collinearity, which indicated that the *U. reniformis* genome presents a mosaic structure in comparison to *U. gibba* (Figure 3A,B). Approximately 114 Mb of the *U. reniformis* genome exhibited partial matches (average length of 84-Kb) to *U. gibba* (Table S4). The unaligned blocks corresponded to the segments exclusive to *U. reniformis*, which were mostly related to the TE-related regions (Figure 3B).

Additionally, we mapped less than 37% of the *U. reniformis* trimmed paired-end reads consistently with the right distance and orientation onto the *U. gibba* assembly, which also supported the considerable structural differences between these species (Table S5). As a result, it was not possible to employ the reference-assisted assembly procedure to anchor the *U. reniformis* scaffolds into the *U. gibba* fully-assembled chromosomes to reconstruct potential pseudo-molecules.

Moreover, in comparison to other angiosperms, *U. reniformis* also showed a lower percentage of macrosyntenic blocks. For instance, *U. reniformis* retained at least 47 Mb (average length of 76-Kb and ANI of 82%), 35 Mb (average length of 46-Kb and ANI of 76%), and 53 Mb (average range of 63-Kbp and ANI of 77%) of shared blocks to *A. thaliana*, *V. vinifera*, and *S. lycopersicum*. In contrast, its closest relative, *U. gibba,* retained 71 Mb (average length of 44-Kb and ANI of 80%), 57 Mb (average length of 26-Kb and ANI of 75%), and 58 Mb (average length of 35-Kb and ANI of 77%) blocks, respectively (Table S4).

**Figure 4:**
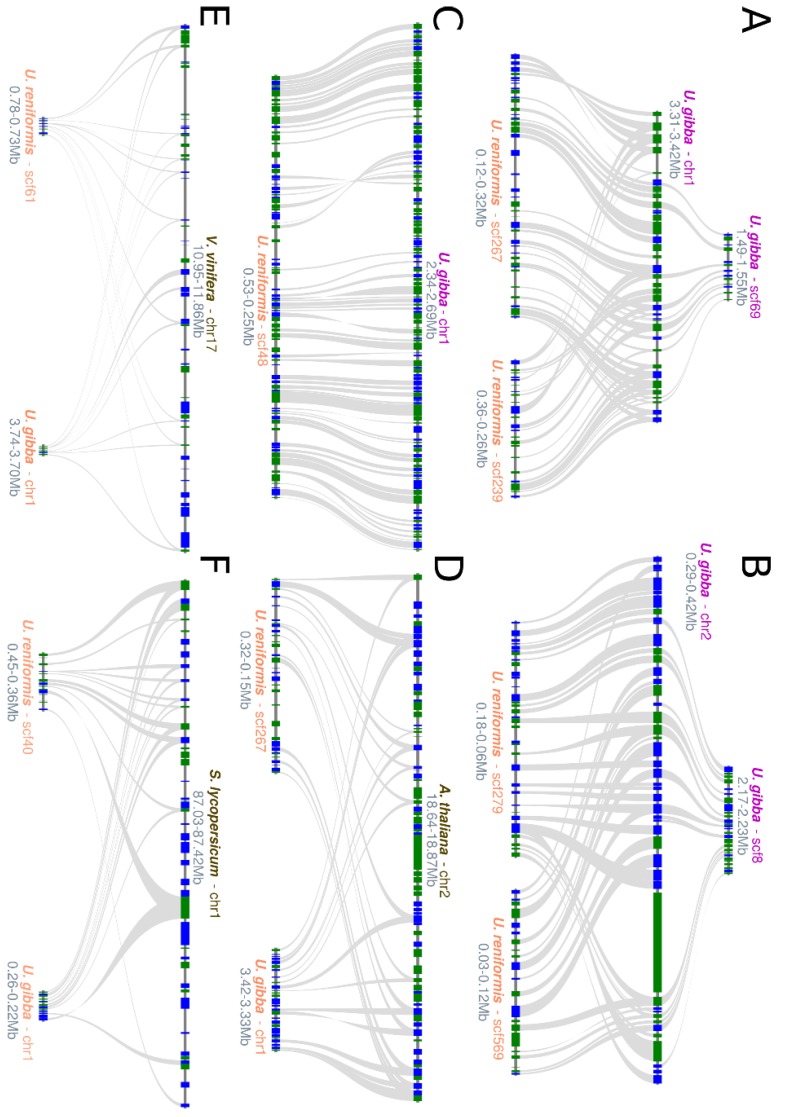
Microsynteny analysis of *Utricularia reniformis* against *U. gibba* and other eudicotyledons: (**A**,**B**) Polyploid subgenomes of *U. reniformis* vs. *U. gibba* microsynteny showing an alternative deletion of the duplicated genes. (**C**) Highly conserved regions of *U. reniformis* vs. *U. gibba* microsynteny. (**D**) *Arabidopsis thaliana* vs. *U. reniformis*, with *U. gibba* showing moderately conserved regions of microsynteny. (**E**) *Vitis vinifera* vs. *U. reniformis*, with *U. gibba* showing a considerable fractionation. (**F**) *Lycopersicon esculentum* vs. *U. reniformis*, with *U. gibba* showing a moderate fractionation.

We further evaluated the microsyntenic level and verified that *U. reniformis* and *U. gibba* present distinct patterns of retention and alternative deletion of duplicated genes between the polyploid subgenomes, which is consistent with the distinct post-speciation evolutionary paths (Figure 4A–C). This feature is also observed among the *Utricularia* species and *A. thaliana*, yet with fewer microsynteny blocks (Figure 4D). When both *Utricularia* genomes are compared to the phylogenetically distant eudicotyledons, such as *V. vinifera* and *S. lycopersicum,* a significant fractionation is prominent (Figure 4E,F).

**Figure 5:**
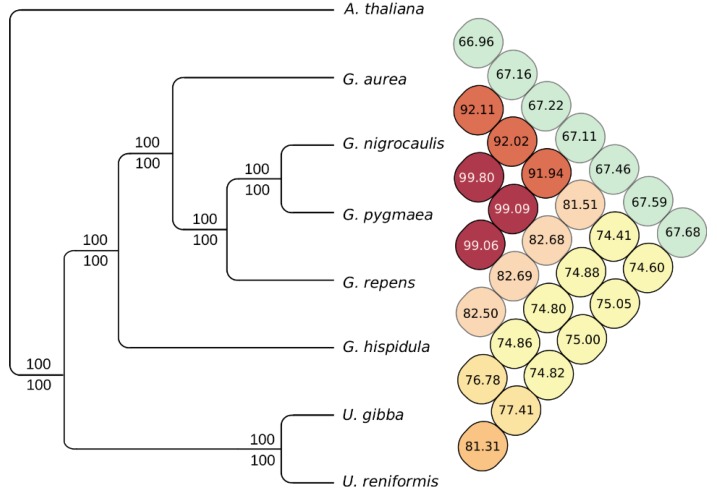
Overall similarity heatmap and maximum likelihood and Bayesian inference phylogenetic tree of 336 core-eukaryotic genes shared among seven carnivorous plant genomes (*Utricularia reniformis*, *U. gibba*, *Genlisea aurea*, *G. nigrocaulis*, *G. pygmaea*, *G. repens*, and *G. hispidula*) and *Arabidopsis thaliana* as an out-group. Numbers above and below the lines indicate the maximum likelihood bootstrap values and the Bayesian posterior probabilities for each clade. The *G. pygmaea* and *G. repens* genomes used in this analysis are yet to be published. However, the concatenated core nucleotide gene fasta sequence used to construct this dataset is freely available at http://doi.org/10.5281/zenodo.3268745.

However, in general, the two *Utricularia* species were far more similar in gene order to each other than either were to *A. thaliana*, *V. vinifera*, and *S. lycopersicum*. The phylogenetic and ANI analyses based on 336 core eukaryotic genes corroborated our microsynteny analysis, supporting the same common ancestor for *U. reniformis* and *U. gibba*, and also for the *Utricularia* and *Genlisea* clades (Figure 5).

### 2.3. Utricularia reniformis Comparative Annotation

In general, *U. reniformis* and *U. gibba* show different arrays of gene duplication that might directly reflect support that they are retaining genes in a biased manner. This was exemplified by the more significant number of tandem, proximal, and dispersed gene pairs identified in *U. reniformis*, when compared to *U. gibba* (Table 3). In contrast, *U. gibba* displayed more singletons and segmental duplicates. However, the more significant number of segmental copies identified in *U. gibba* might be related to the assembled genome using long-reads, which permitted a better identification of large syntenic blocks, in comparison to the *U. reniformis* genome based on short-reads. Moreover, the considerable number of singletons in *U. gibba* might also represent retained duplicates that have reverted to a single copy after the WGD, which supported the genome size reduction observed in this species.

It is noteworthy that the gene space in *U. reniformis* represents ~26% of the genome, which also demonstrated that the intergenic regions are replete with TEs-related sequences constituting more than half of the genome size. Conversely, TEs covered ~32% of *U. gibba* genome, while the gene space represented ~51%.

### 2.4. Utricularia reniformis Shows a Massive Expansion of LTR from the Gypsy Superfamily

**Figure 6:**
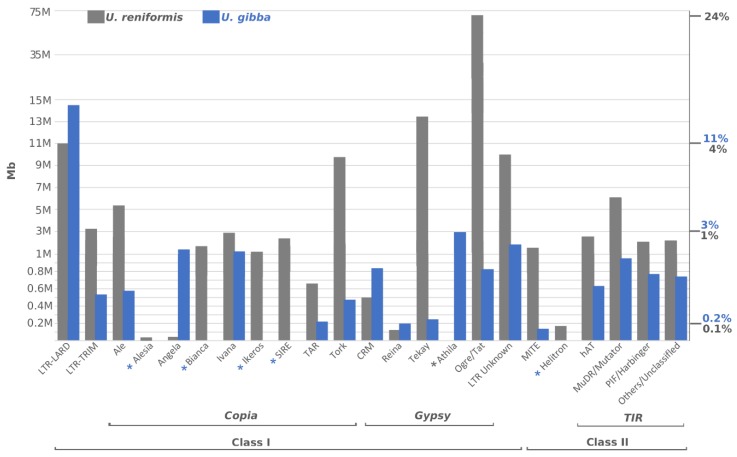
Transposable elements discovery in *Utricularia reniformis* and *U. gibba* genomes: Length occupied (bp) of each super-family and evolutionary lineage. A detailed distribution of each element identified is presented in Table S6. The *y*-axis on the left indicated the total length occupied in the megabases of each evolutionary lineage, whereas the *y*-axis on the right meant the % of the corresponding genome. Blue and Grey asterisk correspond to the absent evolutionary lineages in *U. gibba* and *U. reniformis*, respectively.

*Utricularia reniformis* presents a prominent LTR-retrotransposons expansion, representing up to 145 Mb (Figure 6 and Table S6). In general, a separate array of expansion and contraction of distinct evolutionary lineages from the *Copia* and *Gypsy* superfamilies stand as the main differences between *U. reniformis* and *U. gibba*. The most noticeable increase of the *Gypsy* superfamily was observed within the *Ogre* evolutionary lineage, accounting up to 72 Mb of *U. reniformis* genome. It is noteworthy that the LTR-LARDs, *Angela*, *CRM*, are more prevalent in *U. gibba*, including the *Athila* evolutionary lineage, which was exclusively found in *U. gibba.* In contrast, from the *Copia* superfamily, the evolutionary lineages *Alesia, Bianca, Ikeros*, and *SIRE* were found solely in *U. reniformis.* As observed in other plant genomes, the Class II elements were less prominent in both species, and except for the *Helitron* super-family which we were not able to identify in *U. gibba*, mostly known super-families and evolutionary lineages commonly found in plant genomes were present.

### 2.5. Distinct Functional Enrichment Patterns Among Tandem and Dispersed Duplicate Genes

Self–self-genome comparisons among the singletons and duplicated genes (dispersed, proximal + tandem, and segmentals) revealed exciting patterns of enriched GO terms and KEGG enzymes (*p* < 0.05, Fisher’s exact test, Bonferroni corrected) between *U. reniformis* and *U. gibba* (Tables S7–S9). Both species retained a conserved arrangement of cellular components related to organelles and nucleus among the singletons, such as, chloroplast (GO:0009507), thylakoid (GO:0009579), mitochondrion (GO:0005739), nucleoplasm (GO:0005654), nuclear envelope (GO:0005635), endoplasmic reticulum (GO:0005783), and biological processes related to photosynthesis (GO:0015979) and DNA metabolic process (GO:0006259). Additionally, KEGG enzymes with associated functions that act on NADH or NADPH (EC:1.6.99.5), NADH dehydrogenase (EC:1.6.99.3), NADH—ubiquinone reductase (EC:7.1.1.2) were also augmented among the singletons. Together, these findings support a conserved organellar functional activity including the control of energy homeostasis functions among the singletons.

Among the dispersed duplicates, the nuclease activity (GO:0004518) was enriched in both species. However, the hydrolase activity (GO:0016787), transcription regulator activity (GO:0140110), DNA-binding transcription factor activity (GO:0003700), and tropism (GO:0009606) were significantly overrepresented in *U. reniformis*, while carbohydrate metabolic process (GO:0005975), transport (GO:0006810), kinase activity (GO:0016301), and lipid metabolic process (GO:0006629) were enriched in *U. gibba*.

It was previously observed in many angiosperm genomes that secondary metabolic function and transcriptional function are enriched among tandems and segmental duplicates, respectively [5,28,29,30]. Our results corroborate this observation for secondary metabolic function in the tandem repeats of both *Utricularia,* and the transcriptional functions only in *U. gibba* segmental duplicates. Interestingly, and opposite to what has been previously observed, the transcriptional features were augmented in *U. reniformis* dispersed duplicates genes. Since the dispersed copies could arise from TEs movements, and the *U. reniformis* genome had a high level of LTR-elements, this result might suggest a central role of LTR transposition in molding these genomic differences.

**Figure 7:**
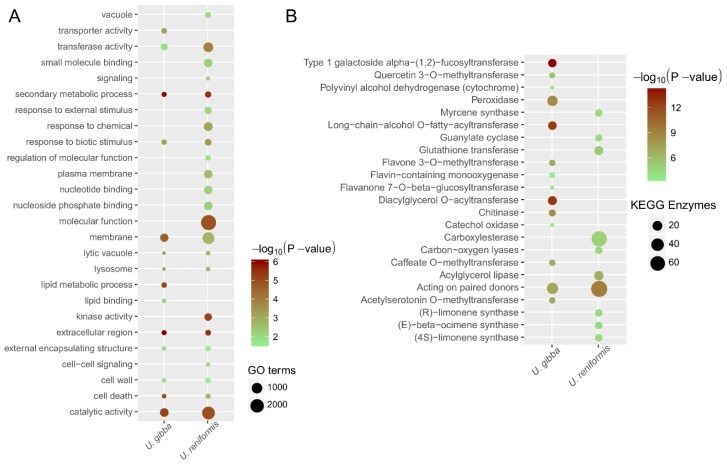
Functional enrichment comparative analysis of tandem duplicates among *Utricularia reniformis* and *U. gibba*: The enriched GO terms and KEGG enzymes with the corrected *p*-value < 0.05 are presented. The color of the circle represents the statistical significance of the enriched GO terms (**A**) and the KEGG Enzymes (**B**). The size of the circles represents the number of occurrences of a GO term (**A**) and the KEGG Enzyme (**B**).

We also checked the potential role of tandem and proximal duplicates with the evolution of novel functions, such as those related to carnivory and the ROS metabolism, which revealed an enrichment of many GO terms and KEGG enzymes in both species (Figure 7). For instance, catalytic activity (GO:0003824), biotic stimulus (GO:0009607), transferase activity (GO:0016740), and the cellular components—lysosome (GO:0005764), lytic vacuole (GO:0000323), and the cell wall (GO:0005618) were overrepresented. The enrichment of the cell wall (GO:0005618) term, could be related to the trap movement during the prey capture, which demands profound and dynamic cell-wall changes [5], and the lysosome and lytic vacuole enrichment might be related to the digestion of preys. However, we also identified distinct GO and KEGG enrichment arrangement among species, strongly suggesting a functional divergence, subfunctionalization, or neofunctionalization among the tandem and proximal duplicates (Figure 7). Comparatively, *U. reniformis* showed an enrichment of response to chemicals (GO:0042221), a response to external stimulus (GO:0009605), and signaling (GO:0023052), whereas, as observed in the dispersed duplicates, the lipid metabolic process (GO:0006629) was also augmented in *U. gibba* tandem duplicates. Regarding the well-known carnivory [4,5] and ROS enzymatic functions, *U. gibba* presented chitinase (EC:3.2.1.14), cysteine protease (EC:3.4.22), and peroxidases (EC:1.11.1.7) enriched in tandem duplicates regions, as previously described [5], whereas *U. reniformis* exhibited glutathione transferases (EC:3.4.16), carboxylesterase (EC:2.5.1.18), acylglycerol lipase (EC:3.1.1.23), pectinesterase (EC:3.1.1.11), and enzymes that acted on the paired donors, with an incorporation or reduction of molecular oxygen (EC:1.14.13). Therefore, these findings indicated that both species presented distinct enrichment patterns of GO terms and KEGG enzymes, supporting an ongoing gene birth–death–innovation process that included metabolism and carnivory-associated functions, among the tandem and proximal duplicated genes.

Interestingly, among the segmental duplicates, similar patterns of functions related to the primary metabolism, and the plant developmental process were enriched in both species, for example the amide biosynthetic process (GO:0043604), anatomical structure development (GO:0048856), reproduction (GO:0000003), nitrogen compound metabolic process (GO:0006807), peptide metabolic process (GO:0006518), and the primary metabolic process (GO:0044238).

### 2.6. Utricularia reniformis Displays Unique Patterns of Carnivory-Associated, Land Adaptation, and Developmentally Related Genes

**Figure 8:**
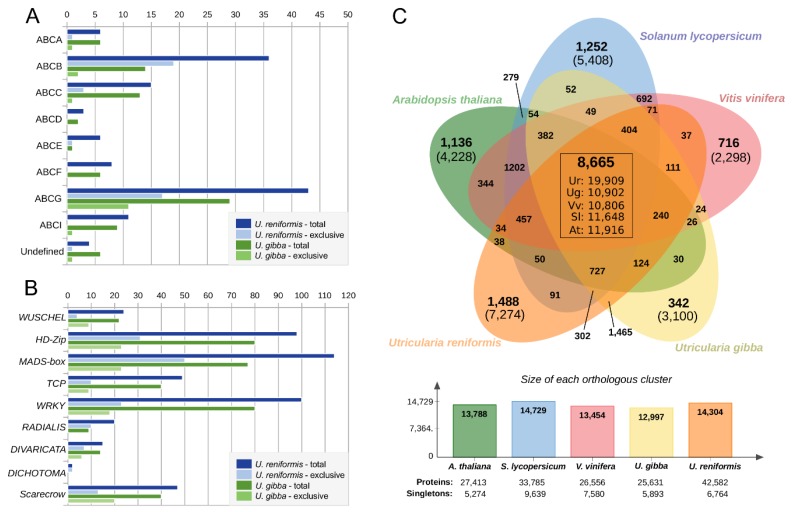
(**A**) Distribution of ABC transporters and (**B**) developmental-related transcription factors among *Utricularia reniformis* and *U. gibba*. Exclusive genes are species-specific genes for which no orthologs could be found in any of the other species compared. (**C**) Venn diagram showing the distribution of shared orthologous gene families among *U. reniformis, U. gibba,* *Arabidopsis thaliana, Vitis vinifera,* and *Lycopersicon esculentum* (using an MCL inflation factor of 1.5). The values present inside the box correspond to the number of core genes of each species. The number present inside the parentheses represent the total number of species-specific genes from each species cluster.

Previous studies, including literature review and comparative annotation approaches, accurately identified GO terms and KEGG enzymes strictly related to the carnivory-associated functions among the sequenced carnivorous plants available in public databases [31,32]. Based on this data, we investigated our functional annotation results and observed that *U. reniformis* had an extensive gene repertoire of carnivory-associated and ROS-related functions (Table 4). In addition, we also identified an extensive repertoire of plant development, reproduction, and life-form adaption GO terms in *U. reniformis* (Table 5), which might be related to the different life-forms in comparison to *U. gibba*. Moreover, distinct patterns of ABC transporters were observed between *U. reniformis* and *U. gibba* (Figure 8A,B). Terrestrial plants exhibited, on average, a repertoire of 128 ABC transporters, while the aquatic plants had fewer copies [33]. At least 132 and 86 ABC transporters were identified in *U. reniformis* and *U. gibba*, respectively (Figure 8A and Table S10), suggesting that *U. reniformis* had more copies due to its terrestrial adaptation. This was especially apparent from the expansion of the ABC transporter B (Figure 8A and Figure S4) and ABC transporter C families, which are commonly related to developmental processes and functions necessary for life on dry land, and the ABC G family (Figure 8A and Figure S5), which are related to response to biotic stress [33]. Furthermore, plant developmental-related transcription factor (TFs) containing the wuschel-like homeobox (WOX), homeobox-leucine zipper (HD-Zip), agamous-like (MADS-box), TCP, WRKY, RADIALIS, DIVARICATA, DICHOTOMA, and the scarecrow-like gene families present distinct patterns of expansion and contraction among both species (Figure 8B and Tables S11–S14), and thus, provide support for the two distinct life-forms between these species.

### 2.7. Comparative Annotation Among Other Angiosperms Genomes Reveals Species-Specific Genes Strictly Related to the Environment and Life-Form Adaptations

The comparative orthologous gene cluster analysis revealed that approximately 60% of *U. reniformis* and *U. gibba* genes are shared, indicating that they were inherited from the last common ancestor. Interestingly, *U. reniformis* presents an almost duplicated core gene set from that observed in *U. gibba*, *V. vinifera*, *A. thaliana*, and *S. lycopersicum*, and thus, supports a species-specific WGD event (Figure 8C and Table S15). In general, a significant fraction of the species-specific genes (genes for which no orthologs could be found in any of the other species compared) and single-copy gene clusters from *U. reniformis* and *U. gibba* encode uncharacterized or hypothetical proteins (63% for *U. reniformis* and 50% for *U. gibba*). Among the species-specific genes, we highlighted ABC transporters, and several developmental-related TF gene families (Figure 8A,B), supporting the role of the species-specific genes with plant development, reproduction, prey strategies, and life-from adaptations. GO enrichment analysis (*p* < 0.05, Fisher’s exact test, Bonferroni corrected) among the species-specific genes revealed an augmented functions related to the reproductive process (GO:0022414), hydrolase activity (GO:0016788), nuclease activity (GO:0004518), catalytic activity (GO:0003824), protein binding (GO:0005515), and organelles (GO:0043226) in both species. However, *U. reniformis* present a unique enrichment of kinase activity (GO:0016301), transferase activity (GO:0016740), carbohydrate metabolic process (GO:0005975), lytic vacuole (GO:0000323), and lysosome (GO:0005764), whereas, *U. gibba* exhibited cellular nitrogen compound metabolic process (GO:0034641), peptide metabolic process (GO:0006518), and signal transduction (GO:0007165). Taken together, these results indicate that the species-specific gene set might play an essential role in the diversification and adaptation to different life-forms.

## 3. Discussion

### 3.1. The Role of WGD in Utricularia reniformis Genome Evolution

Polyploid occurred multiple times during angiosperms evolution, and this is not an exception for *Utricularia* lineages. For instance, multi-way microsynteny analysis revealed three sequential WGD events in *U. gibba*, in addition to the γ WGT event shared by all core eudicots [4,5,34]. Additionally, out-crossing events might be broad among the *Utricularia* clade, and a comparative analyses revealed that the most recent *U. gibba* WGD was derived from an allopolyploidization event [5]. Allopolyploidization is recognized as a driving evolutionary innovation and is often associated with speciation when resulting in a duplicated genome structure [35,36,37]. Therefore, the current hypothesis is that not only was the *U. gibba* genome shaped by WGD events, but other *Utricularia* genomes and specifically the *U. reniformis* genome were shaped by WGD-induced variation and adaptation.

After a polyploidy event, each lineage undergoes distinct patterns of gene retention and fractionation, which generally leads to the genome returning to the diploid state [38]. Our results for *U. reniformis* and *U. gibba* support that each species has distinct patterns of deletions, duplicated genes, and rearrangements, suggesting that after speciation and WGD, they took distinct evolutionary paths that were consistent with their life-histories. This is exemplified by the significant number of tandem, proximal, and dispersed gene pairs identified in *U. reniformis*, when compared to *U. gibba*. Indeed, gene duplication is recognized as one of the major sources of evolutionary innovation [39]. For instance, tandem and proximal duplicated genes are often associated with the emergence of functional novelty, which commonly originated from isolated events in which an individual gene gets duplicated by unequal crossing-over between similar alleles [39]. Together with a large number of dispersed duplicates identified, the tandem duplicated genes might also arise by translocations mediated by TEs [39], generating single-gene transposition-duplication, and therefore, indicating for an essential role of TEs molding the genome structure and evolution. Additionally, WGD analysis confirmed that *U. reniformis* experienced at least one WGD round since the core eudicot WGT. However, it should be taken into account that the low assembly contiguity based on short-read technology hindered the identification of large syntenic blocks, and thus, complicated the evaluation of the complete history of *U. reniformis* WGD events.

### 3.2. LTR-Retrotransposons are Key Agents Governing Utricularia Genome Size Changes

One of the most common repetitive content of *U. reniformis* is related to TEs-related sequences, corresponding to at least 56% of the genome. The TEs drive the genome evolution, mainly by rearrangements, gene promoters repression, enhancers disruption, epigenetic regulation, and also by inflating the genome size by its copy-and-paste mechanism [40,41]. Apart from their deleterious effects, TEs also provide genome variation that can lead to the plant adapting more rapidly to new environmental conditions, and thus, leading to speciation [42,43]. The increased number of TEs in *U. reniformis* vs. *U. gibba* suggests that TEs might be playing a role in the genome innovation we are observing, but it could also just reflect a difference in the ability of the two genomes to purge mobile TEs.

The most prominent TE expansion in *U. reniformis* is related to LTR-retrotransposons elements from the *Gypsy* superfamily, which was also observed in the Solanaceae and Brassicaceae families [44,45]. In particular, elements from the *Tat* lineage were the most prominent, which are commonly broader in other plants, constituting up to 40% of the genome of some species [46].

Except for LTR-LARDs, all other TE groups are expanded in *U. reniformis*. The LTR-LARD and *CRM* are generally located in complex and highly repeated genomic loci, such as heterochromatic and pericentromeric regions [47,48]. These regions are often partially assembled using short-read technology, and we verified that most LTR-LARDs identified in *U. reniformis* are incompletely assembled, suggesting that they were partially determined and underestimated. Therefore, LTR-LARD and *CRM* elements might potentially represent a considerable fraction of the genome that remains unresolved in the *U. reniformis* assembly, and the results presented might be biased due to the assembly contiguity limits.

### 3.3. The Genomic Landmarks for Terrestrial Adaptation

Adaptation to the environment and life-form plasticity are hallmarks of the selective pressures that govern genome evolution in the *Utricularia* lineage. A common reproductive strategy for several *Utricularia* is to produce clones by stolon fragmentation, which is a usual asexual reproduction of aquatic species from the section *Utricularia* (the section in which *U. gibba* is nested)*,* with some species being sterile and rarely flowering [49,50]. In contrast, *U. reniformis* presents a specialized sexual reproductive strategy that is entirely dependent on pollinators, and is specifically dependent on self-pollination [51]. Moreover, *U. gibba* seems to have a more severe degree of Fuzzy Arberian Morphology, such as no clear delimitation of distinct vegetative organs [52]. In contrast, *U. reniformis* presents a more traditional vegetative organ delimitation (as stems and leaves), similar to other angiosperms.

Consequently, the vegetative plasticity found in different *Utricularia* species might be essential strategies used to colonize the distinct habitats. Considering the different environments (water, soil, rock surface, etc.), the various trap designs [53,54,55] might be shaped as adaptation for different prey faunas [56,57,58,59]. Ultimately this might impact the absorption and assimilation of nutrients. At the genomic scale, both species display distinct patterns of development-related genes and TFs, such as WOX, HD-Zip, MADS-box, WRKY, TCP, RADIALIS, DIVARICATA, DICHOTOMA, and scarecrow-like gene families. These TFs play specialized roles in plant growth and development by regulating cell division and differentiation, in particular, the trap and floral meristem and trichome development, and might also develop an important role in the regulation of flowering time and the ontogeny of reproductive organs [16,60,61,62,63,64]. For instance, the WOX1 (3 vs. 5 species-specific genes) from the WOX family, is possibly associated with the trap development [16] SOC1 (11 vs. 6 species-specific genes) from the MADS-box family, which might develop a role with phosphorus scavenging from the trapped prey [4], and ATHB51 (5 vs. 6 species-specific genes) related to the leaf morphogenesis. These findings are suggestive to a different array of target genes, and the processes that these TFs control, and in addition to the distinct number of developmental genes identified in both species, might apply in different body plan adaptations and prey strategies.

Moreover, both species display a distinct repertoire of ABC transporters that are considered essential to terrestrial life-form adaption [33]. Some ABC transporters members are exclusively found in *U. reniformis*. For instance, the ABCB member 9 is related to plant hormone transport, regulation of growth, and in development [65]. Additionally, the mitochondrial ABCB family member 25 was directly involved with the molybdenum cofactor biosynthesis, and nitrogen assimilation from the soil [66]. Interestingly, aquatic plants mainly rely on ammonium as their primary nitrogen source, and the molybdenum cofactor biosynthesis deficiency is commonly associated with the use of ammonium as an alternative nitrogen source in habitats with increased relative humidity [66]. This suggests that the absence of ABCB members 25 in *U. gibba* might be directly related to their aquatic adaptation. Finally, the ABCG member 36, exclusively found in *U. reniformis*, is related to the export of related defence compounds and callose deposition at the site of pathogen contact to restrict pathogen development [67,68], supporting that *U. reniformis* needs extra protection for cellular damage, which is a common characteristic of terrestrial plants.

It is known that carnivorous plants experience different and variable levels of nutritional stress [69], and the emergence of the carnivory-associated function in each lineage was a result of multiple evolutionary paths [31]. Interestingly, *U. reniformis* presents a distinct and augmented set of well-known carnivory-associated functions. Among them, alpha-galactosidase, amidase, actin filament, aspartic-type endopeptidase, beta-galactosidase, cellulase, chitinase and cysteine-type peptidase, myosin heavy chain kinase, and polygalacturonases might be related to carbon and energy cycles maintenance with cellulose and its associated polymers-decomposing activity, suggesting a potential role in the breakdown of prey polysaccharides. Moreover, the enrichment of ATPase activities might be correlated with the trap acidity and molecular transport functions, such as the release of digestive enzymes and the absorption of digested material from the preys [5,31,70].

Conversely, in addition to carnivory-associated functions, the ROS-related enzymes are also augmented in *U. reniformis*. For instance, catalase, superoxide dismutase, peroxidase, and glutathione transferase might contribute to cellular homeostasis and impacting the control of the high respiration rates possibly originated through the carnivory process. In addition, *U. reniformis* exhibits much more dispersed, tandem, and proximal duplicated genes. The emergence of tandem duplicated genes might contribute to the evolution of novel functions and adaptation. Our results provide support for an ongoing gene birth–death–innovation, occurring mainly among tandem and dispersed duplicates, impacting a number of different GO terms and KEGG enzymes, including carnivory-associated functions across both *Utricularia*. This process might give a fine-tuning of carnivory-associated functions in each selective environment (aquatic and terrestrial). For instance, a high diversity of bacteria, fungi, algae, and protozoa compose the ecosystem inside the *Utricularia* trap and act synergistically to convert complicated organic matter into a nutrients source for the plants [71]. The trap ecosystem and prey biota can significantly vary between the terrestrial and aquatic habitat. The aquatic *Utricularia* species usually have a prey spectra dominated by copepodids, cladocerans, ostracods, rotifers, and aquatic insect larvae, while terrestrial species can also have acari, nematodes, and other terrestrial microorganisms [56,58,69,72,73]. Moreover, the environment and the capacity to capture preys can be determined by the trap and prey size [74]. Thus, the trap size difference between the two species (*U. gibba* possess traps c. 0.7–1.5 mm and *U. reniformis* c. 1–2.5 mm long; [1]) can also be the result of selection pressure for the different adaptation to aquatic (*U. gibba*) and terrestrial and epiphytic life-forms. Therefore, both plants might present a distinct trap ecosystem and an enzymatic digestive repertoire to uptake nutrients, and our results supported this hypothesis at a genomic scale. However, this hypothesis warrants further functional studies for confirmation.

## 4. Materials and Methods

### 4.1. Plant Material, Genome Size Estimation, and Cytogenetic Analysis

*Utricularia reniformis* samples were collected in the fall of 2015 in the Serra do Mar Atlantic Forest, in the Municipality of Salesópolis, SP, Brazil, and were deposited in the Herbarium JABU at the São Paulo State University (voucher—V.F.O. de Miranda et al., 1725). The plants were grown in a jar culture before procedures. These samples were recorded in the Brazilian National System of Management of Genetic Heritage and Associated Traditional Knowledge (SisGen) under the access number #A68D114, in accordance with the Brazilian Access and Benefit Sharing (ABS) legal framework introduced by the Federal Law No. 13,123 of 2015. No permission for collecting was necessary, as the sample was not collected in protected areas and *U*. *reniformis* is not a threatened species, according to the global IUCN (The IUCN Red List of Threatened Species: http://www.iucnredlist.org) and the Brazilian List of Threatened Plant Species.

For the genome size estimation, approximately 25 mg of leaf tissue was macerated in 1 mL of cold Ebihara buffer [75] supplied with 0.025 µg mL^−1^ RNAse, using a scalpel blade to release the nuclei into suspension with the same mass of the internal reference standard *Raphanus sativus* var. Saxa (2C = 1.11 pg; [76]). The nuclei suspensions were stained by adding 25 µL of a 1 mg mL^−1^ solution of propidium iodide (PI, Sigma). The analysis was performed using the FACSCanto II cytometer (Becton Dickinson, San Jose, CA, USA). The histograms were obtained through the FACSDiva software based on 5000 events, and the statistical evaluation was performed using the Flowing Software 2.5.1 (http://www.flowingsoftware.com/). Three individuals were analyzed in triplicates. The quality control of samples was based on the coefficient of variation (CV) of each measurement (which should be below 5%) and the standard deviation (SD) among the 2C-values (which should be below 3%). Such limits ensure that the variations observed inside and among measurements are due to technical factors and do not represent intraspecific variation among individuals [77].

For the cytogenetic analysis, stolon tips were pre-treated in 8-hydroxyquinoline (0.002 M) for 24 h, at 10 °C, fixed in ethanol:acetic acid (3:1, *v/v*) for 24 h, at room temperature, and were stored at −20 °C. The fixed stolon tips were washed in distilled water and digested in a 2% (*w/v*) cellulase (Onozuka)/20% (*v/v*) pectinase (Sigma-Aldrich, St. Louis, MO, USA)/1% macerozyme (Sigma-Aldrich, St. Louis, MO, USA) solution, at 37 °C for 15 min. The meristems were squashed in a drop of 45% acetic acid, and the coverslip was later removed in liquid nitrogen. Metaphases were DAPI (1 µg·mL^−1^) stained and photographed with an XM10 camera coupled to a BX 53 Olympus epifluorescence microscope.

### 4.2. Genome and Transcriptome Sequencing

One individual was sampled, producing the three different DNA libraries used in this study. DNA was extracted using the QIAGEN DNeasy Plant Maxi Kit extraction protocol (Qiagen, Hilden, Germany) to prepare libraries for Illumina TruSeq PCR-free and Nextera XT paired-end reads, with ~350 bp (2 × 100 bp) and ~450 bp (2 × 300 bp), insert sizes, and Illumina Nextera mate-pair gel free with insert sizes ranging from 3.5 to 10 Kb (2 × 100 bp). Illumina libraries were sequenced on HiScanSQ and MiSeq machines. The paired reads (2 × 100 bp and 2 × 300 bp) adapters were removed with Trimmomatic v0.27 [78], while the mate-pair reads (2 × 100 bp) internal adapters were trimmed with the NxTrim [79]. Sequences with phred value below 24 (<Q24) and spanning less than 50 bp were removed. For transcriptome sequencing, we used samples from leaves, stolon, and utricles. The RNA extraction and sequencing was performed according with the *U. reniformis* previous research [26,27]. Trimmed RNAseq reads from the leaves, stolon, and utricles were concatenated, and de novo assembled using Trinity v2.7.0 [80], and were exclusively used for gene model predictions (see below).

### 4.3. Genome Assembly and WGD Analysis

The trimmed reads were filtered against the cpDNA (NC_029719) and mtDNA (NC_034982) organellar genomes [26,27] with bowtie2 v2.26 [81]. The assembly was carried out with MaSuRCA v3.1.3 [82], SSPACE [83], and GapCloser [84]. REAPR v1.0.18 [85] was used to generate the corrected assembly and estimate the percentage of error-free bases (additional details are provided in Supplementary Materials). CEGMA v2.5 [86] and BUSCO v3 [87] using Eudicotyledons_odb10 from the OrthoDB database [88] were used to measure the genome completeness. We used GenomeScope v1 [89], the K-mer Analysis Toolkit (KAT) [90], and *KmerSpectrumPlot.pl* from ALLPATHS-LG [91] to estimate genome size and heterozygosity.

The predicted WGDs events that occurred in the *U. reniformis* evolutionary history were predicted on the basis of the distribution of synonymous substitutions per synonymous site (Ks) of gene pairs, using the SynMap2 tool from the Comparative Genomics Platform (CoGe) [92]. MCScanX [93] was employed to identify dispersed, proximal, and tandem (small-scale duplication) and segmental duplicates (WGDs). Tandem duplicates are defined as paralogs that are adjacent to each other, and the maximum distance to call a proximal copy was set to 10 genes. The anchor genes in collinear blocks inferred segmental duplicates. Structural and comparative analyses (pairwise alignment) were carried out with D-GENIES [94]. Macrosynteny and microsynteny analyses were carried out with VGSC2 and MCscan (Python version) (https://github.com/tanghaibao/jcvi.wiki.git).

### 4.4. Transposable Elements Identification, Classification, and Genome Annotation

The de novo detection and classification of TEs were carried out by the REPET v2.5 [95] with the “—struct” parameter. The identified elements from the REPET package were manually validated and characterized into super-families and evolutionary lineages with the usage of the Domain-based ANnotation of Transposable Elements (DANTE) with the Viridiplantae database v3.0 [96]. The TE masking was performed with the RepeatMasker Open-v4.0.7 (http://www.repeatmasker.org) using the parameter: “-s -cutoff 260”.

The gene prediction was performed using the BRAKER v2 pipeline [97], and the TE-related genes were discarded. PASA pipeline [98] was used to produce spliced alignment assemblies based on RNA-seq data. The gene predictions and transcript alignments were combined with the EVidence Modeler software [99]. Finally, two PASA pipeline iterations were used to update the EVidence Modeler consensus predictions, adding UTR annotations and models for the alternatively spliced isoforms. Functional annotation was performed using Blast2GO v5 [100], InterProScan 5 [101], and EggNOG-mapper v1.0.3 with the EggNOG 5.0 database [102]. For the protein assignment, the UniProtKB/TrEMBL [103] viridiplantae database was used. The final predicted gene models were scanned against the DANTE and RepeatMasker predictions results, and all TE-related genes were further discarded.

For consistency in the comparative analysis, *Utricularia gibba* transcriptomes read from NCBI Sequence Read Archive (SRA): SRX2368915, SRX247091, and SRX038704 were assembled, and the *U. gibba* genome (Genbank Accession Number: NEEC01000001-NEEC01000518) was re-annotated using the same pipeline.

### 4.5. Phylogenetic Analysis

The phylogenetic analyses were performed using maximum likelihood (ML), and Bayesian inference (BI) approaches. For the ML, RAxML v. 8.2.10 [104] was used with default parameters, and the clade support estimates were calculated using rapid bootstrapping of 1000 pseudoreplicates. The BI was performed with Mr. Bayes v.3.2.6 [105] using MCMCMC, with random starting trees, of about 5,000,000 generations, with sampling for each 100 trees, using two runs and four chains and the trees were considered only after a stationary position was reached, with 25% of initially elaborated trees discarded. The GTR + G + I was used as the best-of-fit model according to the AIC (Akaike Information Criterion) with the jModeltest v. 2.1.10 [106].

### 4.6. Comparative Analysis

OrthoVenn v2 [107] was used to determine the orthologous gene families among *Utricularia reniformis*, *U. gibba*, and other angiosperms (*Arabidopsis thaliana, Vitis vinifera,* and *Solanum lycopersicum*) retrieved from the Phytozome database [108]. Functional enrichment analyses of the GO terms and the KEGG enzymes were conducted using Fisher’s exact test and was corrected for multiple testing using two methods employed by the Blast2GO (GO terms and KEGG enzymes) and GOATOOLS (GO terms) [109]. The *p*-values were determined after the correction for multiple tests through False Discovery Rate (FDR) control, according to the Benjamini–Hochberg. The WOX, HD-Zip, MADS-Box Transcription Factors (TFs), and ABC transporters comparative analyses were based on previous [16,33] and manual annotation using the *A. thaliana* reviewed entries from UniProt Database as reference [110].

### 4.7. Availability of Supporting Data

The raw sequencing reads have been deposited into GenBank, BioProject PRJNA290588, linked directly to the SRA under the accession numbers SRR8289569, SRR8289570, and SRR8289571 for the Illumina datasets and SRR8289572 for Ion Torrent’s RNA-Seq data. This Whole Genome Shotgun project has been stored at DDBJ/ENA/GenBank under the accession RWGZ00000000. The version described in this paper is version RWGZ01000000. The gene models and genome browser of *Utricularia reniformis* are available at https://genomevolution.org/coge/GenomeInfo.pl?gid=54799. The Gene Ontology annotation (Blast2GO) and all the raw data generated for this study are freely available at the Zenodo platform at http://doi.org/10.5281/zenodo.3268745.

## 5. Conclusions

In this work, we generated a draft genome sequence and annotation for the terrestrial carnivorous plant *U. reniformis* and compared it against its sister, aquatic species *U. gibba.* Our results demonstrated that a massive proliferation and loss of lineage-specific LTR-retrotransposons, predominantly from the *Gypsy* super-family and WGDs are the main governing agents of the genome size changes and evolution between these species. Interestingly, our results strongly suggest that both genomes responded distinctly after the WGD events. This is mainly observed by specific patterns of gene fractionation and retention after the lineage split, which might be reflected in an ongoing gene birth–death–innovation process among the duplicated genes. Additionally, each species carries a diversified set of plant ontogeny and carnivory-associated gene repertoire, supporting that the reproductive isolation and terrestrial and aquatic life-form constraints (e.g., different nutrients repertoire and availability, prey diversity, and trap microbiome ecosystem) in an evolutionary time scale are associated with speciation.

## Figures and Tables

**Table 1:** DNA and RNA read produced for the *Utricularia reniformis* genome assembly and annotation.

	Technology	Library	Read Length	Raw Reads	Trimmed Reads
DNAseq	Illumina HiScan and MiSeq	Paired-end (~350 bp)	100 bp	285,403,944	187,288,003
		Paired-end (~450 bp)	300 bp	49,185,074	35,963,241
		Mate-paired (3–9 Kb)	100 bp	177,044,066	60,011,867
RNAseq	Ion Proton	Single-end - Leaves - Stolon - Utricules (preys)	~200 bp	46,622,745 41,894,450 112,414,083	40,853,284 34,716,966 97,821,388

**Table 2:** *Utricularia reniformis* genome assembly status.

	*U. reniformis Genome*	
Total size of scaffolds (bp)	304,550,249	
Number of scaffolds	1830	
Number of contigs	5452	
Useful amount of scaffold sequences (≥25-Kb)	297,419,257	
% of assembled genome that is useful	93.8%	
Longest scaffold (bp)	1,862,935	
Longest contig (bp)	926,419	
Scaffolds longer than 1-Kb	1830 (100%)	
Scaffolds longer than 100-Kb	688 (37.6%)	
Scaffolds longer than 1 Mb	47 (2.6%)	
NG50 scaffold length (bp)	466,988	
LG50 scaffold count	196	
N50 contig length (bp)	161,226	
Percentage of assembly in scaffolded contigs	91	
Gaps number	3677	
Unknown bases (Ns) (bp)	5,790,542	
Average gap size (bp)/Longest gap (bp)	1575	10,802

**Table 3:** *Utricularia reniformis* and *U. gibba* comparative and structural annotation.

	*U. reniformis*	*U. gibba*
Total number of identified genes	42,582	25,509
- Singletons	3083	5120
- Dispersed duplicates	26,546	8831
- Proximal duplicates	1679	683
- Tandem duplicates	2994	999
- Segmental duplicates	8280	9876
Annotation status		
- Annotated genes †	35,899	21,283
- Genes with GOs	27,751	17,760
- Unknown and Hypothetical genes	6683	4348
Total gene length (bp)	79,419,967	51,346,373
Total exon length (bp)	44,971,966	28,891,859
Total intron length (bp)	29,871,202	16,875,684
Longest gene (bp)	84,570	61,898
Longest exon (bp)	7309	6002
Longest intron (bp)	78,774	53,378
Longest CDS (bp)	15,201	15,801
Mean gene length (bp)	1872	2016
Mean exons per gene	5	5
% of genome covered by genes	26	51
% of genome covered by CDS	15	38
% of genome covered by TEs-like regions	56	32

† high-confidence genes models (showing a potential product, GO term, and hits to UniProt viridiplantae clade).

**Table 4:** Carnivory-associated and reactive oxygen species (ROS) detoxification GO terms distribution. *Utricularia reniformis* (7200 unique genes: Singletons—3.5%, Dispersed—65.5%, Tandem and Proximal—10.5%, and Segmental—20.5%) and *U. gibba* (3918 unique genes: Singletons—10%, Dispersed—48%, Tandem and Proximal—6%, and Segmental—36%).

Gene Ontology Term	GO Code	*U. renif* n# Genes	*U. gibba* n# Genes
amidase activity	GO:0004040	13	10
actin filament	GO:0005884	20	11
alpha-galactosidase activity	GO:0004557	15	13
alternative oxidase activity	GO:0009916	6	4
ammonium transmembrane transport	GO:0008519; 0072488	18	16
aspartic-type endopeptidase activity	GO:0004190	289	94
ATP:ADP antiporter activity	GO:0005471	9	9
ATPase activity	GO:0016887	1831	792
beta-galactosidase activity	GO:0004565	720	125
catalase activity	GO:0004096	17	11
cellulase activity	GO:0008810	62	34
chitinase activity	GO:0004568	21	13
cinnamyl-alcohol dehydrogenase activity	GO:0045551	34	15
cyclic-nucleotide phosphodiesterase activity	GO:0004112	1	1
cysteine-type peptidase activity	GO:0008234	384	185
nuclease activity	GO:0004518	1955	1094
fructose-bisphosphate aldolase activity	GO:0004332	12	10
glutathione transferase activity	GO:0004364	54	21
glutathione peroxidase activity	GO:0004602	14	9
hydrolase activity, acting on ester bonds	GO:0016788	2767	1259
heat shock protein activity	GO:0042026; 0006986; 0034620	129	72
lipase activity	GO:0016298	231	140
lipid transport	GO:0006869	170	113
myosin heavy chain kinase activity	GO:0016905	36	23
peroxidase activity	GO:0004601	192	140
peptidase activity	GO:0008233	2650	1269
phosphatase activity	GO:0016791	287	186
phospholipase activity	GO:0004620	80	43
polygalacturonase activity	GO:0004650	109	50
polygalacturonase inhibitor activity	GO:0090353	5	1
protein homodimerization activity	GO:0042803	809	525
ribonuclease activity	GO:0004540	390	154
serine-type carboxypeptidase activity	GO:0004185	82	38
superoxide dismutase activity	GO:0004784	17	10
symplast	GO:0055044	1060	679
urease activity	GO:0009039	2	1
water channel activity	GO:0015250	46	26

**Table 5:** Distribution of GO terms related to the biological process related to plant development, reproduction, and life-form adaptation. (*U. reniformis*; 11,468 unique genes: Singletons—3%, Dispersed—62%, Tandem and Proximal—10%, and Segmental—23%. *U. gibba*; 7071 unique genes: Singletons—11%, Dispersed—41%, Tandem and Proximal—4%, and Segmental—43%).

Gene Ontology Term	GO Code	*U. renif* n# Genes	*U. gibba* n# Genes
developmental process involved in reproduction	GO:0003006	1965	1237
reproduction	GO:0000003	2546	1600
reproductive process	GO:0022414	2223	1388
multicellular organism development	GO:0007275	4749	2773
embryo development	GO:0009790	744	465
post-embryonic development	GO:0009791	2447	1578
flower development	GO:0009908	915	601
developmental maturation	GO:0021700	264	171
developmental process	GO:0032502	4126	2704
reproductive structure development	GO:0048608	1685	1065
anatomical structure development	GO:0048856	4046	2639
cellular developmental process	GO:0048869	1352	880
reproductive shoot system development	GO:0090567	655	437
pollination	GO:0009856	343	210
tropism	GO:0009606	215	129
circadian rhythm	GO:0007623	261	161
response to stress	GO:0006950	4513	2869
response to radiation and light stimulus	GO:0009314, GO:0009416	1012	711
response to external stimulus	GO:0009605	2108	1352
response to biotic stimulus	GO:0009607	1526	981
response to abiotic stimulus	GO:0009628	3161	2041
response to endogenous stimulus	GO:0009719	2665	1693
response to chemical	GO:0042221	4590	2984
response to stimulus	GO:0050896	6579	4277

## Supplementary Materials

Supplementary Materials can be found at https://www.mdpi.com/1422-0067/21/1/3/s1.
