# Supplementary material for: The Terrestrial Carnivorous Plant Utricularia reniformis Sheds Light on Environmental and Life-Form Genome Plasticity
Source: Int J Mol Sci. 2019 Dec 18;21(1):3. doi: 10.3390/ijms21010003 (PMC6982007; doi:10.3390/ijms21010003)
Supplement: Supplementary file 1 [file ijms-21-00003-s001.zip › Supplementary_materials.pdf]

International Journal of *Molecular Sciences*

MDPI

Article - Supplementary Materials

The terrestrial carnivorous plant *Utricularia reniformis* sheds light on environmental and life-form genome plasticity

Saura R. Silva[1], Ana P. Moraes[2], Helen A. Penha[1], Maria H. M. Julião[1], Douglas S. Domingues[3], Todd P. Michael[4], Vitor F.O. Miranda[5*] and Alessandro M. Varani[1*]

1 UNESP - Universidade Estadual Paulista, Departamento de Tecnologia, Faculdade de Ciências Agrárias e Veterinárias, Jaboticabal, São Paulo, Brazil.
2 Universidade Federal do ABC, Centro de Ciências Naturais e Humanas, São Bernardo do Campo, São Paulo, Brazil.
3 UNESP - Universidade Estadual Paulista, Departamento de Botânica, Instituto de Biociências, Rio Claro, São Paulo, Brazil.
4 J. Craig Venter Institute, La Jolla, CA, United States of America.
5 UNESP - Universidade Estadual Paulista, Departamento de Biologia Aplicada à Agropecuária, Faculdade de Ciências Agrárias e Veterinárias, Jaboticabal, São Paulo, Brazil.

* Correspondence: A.M.V. alessandro.varani@unesp.br; V.F.O.M. vitor.miranda@unesp.br

Supplementary Materials

Content

Supplementary Information.
Assembly strategy using short-reads technology: Recovering *U. reniformis* genome 2

Supplementary Tables.
Supplementary Table S1 6
Supplementary Table S2 7
Supplementary Table S3 9
Supplementary Table S4 9
Supplementary Table S5 9
Supplementary Table S6 10
Supplementary Table S7 11
Supplementary Table S8 11
Supplementary Table S9 11
Supplementary Table S10 12
Supplementary Table S11 12
Supplementary Table S12 13
Supplementary Table S13 14
Supplementary Table S14 15
Supplementary Table S15 15

Supplementary Figures.
Supplementary Figure S1 16
Supplementary Figure S2 16
Supplementary Figure S3 17
Supplementary Figure S4 18
Supplementary Figure S5 19

Supplementary Information

Assembly strategy using short-reads technology: Recovering *U. reniformis* genome

It is well known the extreme difficulty in assembly polymorphic, polyploidy, repeat-containing, and heterozygous genomes by Illumina short-reads technology [1,2]. This occurs mainly due to the increased complexity of the *de Bruijn* graph structure during the genome assembly [3]. Even using third-generation long-reads sequencing, the assembly of polymorphic genomes is still a challenge [4]. We attempted to assemble *U. reniformis* using the traditional genome assembly software. Among them, ALLPATHS-LG [5], SOAPdenovo [6], Abyss [7], Meraculous-2D [8], Platanus [3] and MaSuRCA [9], failed, resulting in fragmented and miss-assemblies. For instance, ALLPATHS-LG, Meraculous-2D, and MaSuRCA resulted in scaffolds exhibiting gaps longer than any input mate pairs (e.g., gaps with the range from 20-kbps to 500-kbps), and showing total length from 15 to 25-Mbps. The Lentibulariaceae generally exhibits small, bacterial sized chromosomes [10], and our cytogenetic results confirm this tendency for *U. reniformis*; therefore, mostly longer (>15-Mbps) assembled scaffolds might correspond to assembly errors. Another assembly evidence for a polymorphic genome structure was observed during the MaSuRCA scaffold module CGW, which took three months running in a 32 cores machine (Intel(R) Xeon(R) CPU E5-2630 v3 @ 2.40GHz) with 396Gb of RAM. The CGW step loads the entire scaffold graph into the RAM, merging, and breaking scaffolds until it converges to a stable solution [9]. It is well established that long CGW runs are directly related to very large (>20Gb), complex and highly repeated genomes [11]. Therefore, the ALLPATHS-LG, Meraculous-2D, and MaSuRCA failed results are potentially related to genome repetitiveness, which increased the complexity of the scaffold *de Bruijn* graph by the usage of the mate-paired reads. Moreover, the SOAPdenovo, Platanus, and Abyss also generated fragmented genomes assemblies (N50 < 400-bp), spanning between ~500 to 600-Mb, and thus, corresponding to a much larger genome size than flow cytometer predicted genome size (317-Mb). It is also known that highly fragmented short-reads assemblies, showing a total assembled size more extensive than expected genome size is a clear sign of polymorphic genome structure [12]. Taken together, these findings highlight the challenges to assembly of *U. reniformis* genome, evidencing the current limitation of the genome assembly algorithm based on short-reads technology to deal with complex genomes.

To overcome this limitation, in this work we developed a genome assembly approach based in two stages. In the first stage, we employed MaSuRCA assembler due to their capacity to deal with large and complex plant genomes containing complicated repetitive and polymorphic structure, in an attempt to assembly two non- or near-identical repeats or two divergent haplotypes. However, to reduce the complexity of the *de Bruijn* graph structure, and MaSuRCA CGW scaffold step algorithm limitation to deal with sequence repetitiveness and heterozygosity, only high-quality paired-end reads (2x100bp and 2x300bp) were used in the first assembly stage (initial assembly). All sequences (contigs) derived from potential contaminants were manually inspected with blastn v.2.2.30+ [13] and further removed. The initial assembly shows N50 of 13-kbps, and total assembled size of 305-Mb contained in ~59k scaffolds (Table 1, initial assembly).

To improve the assembly contiguity, the initial assembly was further processed (post-processing) to create scaffolds representing the heterozygous haplotypes, by several iterations of SSPACE [14], GapCloser [6], and REAPR [15] (second assembly stage). The trimmed and high-quality mate-paired and paired-end reads were used in the scaffolding step (SSPACE) and only paired-end

reads were used in GapClose stage. The iterations were conducted until no more contigs links were found (containing a minimal five paired-end/mate-paired reads connections consistently with the library size and orientation), and therefore no more new scaffolds were constructed, and no more gaps were filled. The resulting assembly was further processed by REAPR pipeline using all libraries of trimmed reads with the aggressive breaking approach, and the generated scaffolds were again further processed by SSPACE and GapClose iterations, and one last REAPR round. All scaffolds shorter than 500bp were removed from the final assembly. This approach permitted the recovering the final version of *U. reniformis* draft genome assembly (Table 1, post-processing assembly), showing N50 of 466-kbps contained in 1,830 scaffolds. Furthermore, BUSCO [16] analyses revealed 87.8% of genome completeness, in comparison to 88.3% obtained in *U. gibba* genome [17], and the REAPR estimated number of error-free bases is 93% (283 of 304-Mbps), thus, indicating the effectiveness of our assembly approach based on short-reads technology to uncover the biology of the *U. reniformis*, and showing by global pairwise alignments, that *U. reniformis* genome exhibits low global similarity to the *U. gibba* genome (Figure 1).

Table 1. *Utricularia reniformis* genome assembly status before and after the post-processing approach based on scaffold, gap close and REAPR iterations.

	U. reniformis **MaSuRCA initial assembly**		***U. reniformis*** **REAPR/Scaffolds post-processing**	
Total size of scaffolds (bp)	305,013,037		304,550,249	
Number of scaffolds	59,466		1,830	
Number of contigs	59,862		5,452	
Useful amount of scaffold sequences (>= 25-kbps)	91,033,927		297,419,257	
% of assembled genome that is useful	28.7%		93.8%	
Longest scaffold (bp)	170,461		1,862,935	
Longest contig (bp)	170,461		926,419	
Scaffolds longer than 1-kbps	47,008 (79%)		1,830 (100%)	
Scaffolds longer than 100-kbps	13 (0.01%)		688 (37.6%)	
Scaffolds longer than 1-Mbps	-		47 (2.6%)	
NG50 scaffold length (bp)	12,692		466,988	
LG50 scaffold count	6,168		196	
N50 contig length (bp)	13,512		161,226	
Percentage of assembly in scaffolded contigs	2.0%		91%	
Gaps number	5,883		3,677	
Unknown bases (Ns) (bp)	142,622		5,790,542	
Average gap size (bp) / Longest gap (bp)	24	675	1,575	10,802

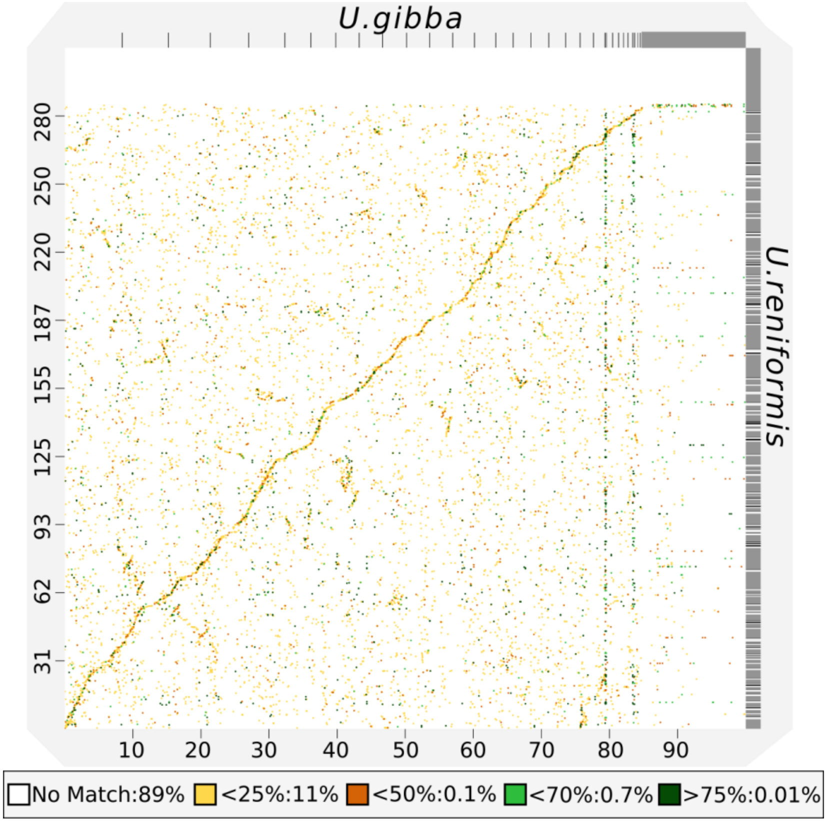

Figure 1. D-GENIES pairwise alignment dot plot showing low global similarity. The overall *Utricularia gibba* vs. *U. reniformis* genome similarity profile, based on the sums of the projections of the matches on *U. gibba* scaffolds similarity values, divided by the total matches of each *U. reniformis* scaffolds, is, in average, less than 25%.

References

1. Richards, S. Full disclosure: Genome assembly is still hard. *PLOS Biol.* **2018**, *16*, e2005894.
2. Schatz, M.C.; Witkowski, J.; McCombie, W.R. Current challenges in de novo plant genome sequencing and assembly. *Genome Biol.* **2012**, *13*, 243.
3. Kajitani, R.; Toshimoto, K.; Noguchi, H.; Toyoda, A.; Ogura, Y.; Okuno, M.; Yabana, M.; Harada, M.; Nagayasu, E.; Maruyama, H.; et al. Efficient de novo assembly of highly heterozygous genomes from whole-genome shotgun short reads. *Genome Res.* **2014**, *24*, 1384–1395.
4. Li, C.; Lin, F.; An, D.; Wang, W.; Huang, R. Genome Sequencing and Assembly by Long Reads in Plants. *Genes (Basel).* **2018**, *9*, 6.
5. Gnerre, S.; MacCallum, I.; Przybylski, D.; Ribeiro, F.J.; Burton, J.N.; Walker, B.J.; Sharpe, T.; Hall, G.; Shea, T.P.; Sykes, S.; et al. High-quality draft assemblies of mammalian genomes from massively parallel sequence data. *Proc. Natl. Acad. Sci.* **2011**, *108*, 1513–1518.
6. Luo, R.; Liu, B.; Xie, Y.; Li, Z.; Huang, W.; Yuan, J.; He, G.; Chen, Y.; Pan, Q.; Liu, Y.; et al. SOAPdenovo2: an empirically improved memory-efficient short-read de novo assembler. *Gigascience* **2012**, *1*, 18.
7. Simpson, J.T.; Wong, K.; Jackman, S.D.; Schein, J.E.; Jones, S.J.M.; Birol, İ. ABySS: A parallel assembler for short read sequence data. *Genome Res.* **2009**, *19*, 1117–1123.

8. Chapman, J.A.; Ho, I.; Sunkara, S.; Luo, S.; Schroth, G.P.; Rokhsar, D.S. Meraculous: De Novo Genome Assembly with Short Paired-End Reads. *PLoS One* **2011**, *6*, e23501.
9. Zimin, A. V; Marçais, G.; Puiu, D.; Roberts, M.; Salzberg, S.L.; Yorke, J.A. The MaSuRCA genome assembler. *Bioinformatics* **2013**, *29*, 2669–2677.
10. Greilhuber, J.; Borsch, T.; Müller, K.; Worberg, A.; Porembski, S.; Barthlott, W. Smallest angiosperm genomes found in Lentibulariaceae, with chromosomes of bacterial size. *Plant Biol. (Stuttg).* **2006**, *8*, 770–7.
11. Zimin, A.; Stevens, K.A.; Crepeau, M.W.; Holtz-Morris, A.; Koriabine, M.; Marçais, G.; Puiu, D.; Roberts, M.; Wegrzyn, J.L.; de Jong, P.J.; et al. Sequencing and Assembly of the 22-Gb Loblolly Pine Genome. *Genetics* **2014**, *196*, 875–890.
12. Pryszcz, L.P.; Gabaldón, T. Redundans: an assembly pipeline for highly heterozygous genomes. *Nucleic Acids Res.* **2016**, *44*, e113.
13. Camacho, C.; Coulouris, G.; Avagyan, V.; Ma, N.; Papadopoulos, J.; Bealer, K.; Madden, T.L. BLAST+: architecture and applications. *BMC Bioinformatics* **2009**, *10*, 421.
14. Boetzer, M.; Henkel, C. V; Jansen, H.J.; Butler, D.; Pirovano, W. Scaffolding pre-assembled contigs using SSPACE. *Bioinformatics* **2011**, *27*, 578–579.
15. Hunt, M.; Kikuchi, T.; Sanders, M.; Newbold, C.; Berriman, M.; Otto, T.D. REAPR: A universal tool for genome assembly evaluation. *Genome Biol.* **2013**, *14*, R47.
16. Simão, F.A.; Waterhouse, R.M.; Ioannidis, P.; Kriventseva, E. V.; Zdobnov, E.M. BUSCO: Assessing genome assembly and annotation completeness with single-copy orthologs. *Bioinformatics* **2015**, *31*, 3210–3212.
17. Lan, T.; Renner, T.; Ibarra-Laclette, E.; Farr, K.M.; Chang, T.-H.; Cervantes-Pérez, S.A.; Zheng, C.; Sankoff, D.; Tang, H.; Purbojati, R.W.; et al. Long-read sequencing uncovers the adaptive topography of a carnivorous plant genome. *Proc. Natl. Acad. Sci.* **2017**, *114*, E4435–E4441.

Supplementary Tables

Table S1. Sporophytic genome size (2C) estimation by flow cytometry. The raw data for the three analyzed individuals, in triplicate, are presented followed by the coefficient of variation (CV %) of each sample and for each repetition of the internal reference standard used Raphanus sativus L. 'Saxa' (2C = 1.11pg). The maximum value accepted for CV is 5% and for standard deviation (SD) is 3%.

Individual	Repetition	2C (pg)	Sample CV (%)	Internal reference standard CV (%)	Sample SD (pg, %)
1	a	0.653	5.00	3.96	
	b	0.661	3.75	4.08	
	c	0.654	4.25	3.16	
2	a	0.645	2.91	3.84	
	b	0.631	3.88	3.49	
	c	0.652	4.75	3.82	
3	a	0.656	4.63	3.5	
	b	0.651	4.59	3.29	
	c	0.649	3.83	3.17	
**Mean**		**0.652**	**4.28**	**3.59**	**± 0.01pg, 1.48%**

Measurements in Mbp	
**Mean 1C**	**0.324**
**Mean 1C in Mbp**	**317.1**

Table S2. CEGMA and BUSCO genome completeness analyses raw results.

(A) CEGMA and BUSCO results of *U. reniformis*

Statistics of the completeness of the genome based on 248 CEGs

	#Prots	%Completeness	#Total	Average	%Ortho
**Complete**	237	95.56	564	2.38	65.40
Group 1	63	95.45	130	2.06	46.03
Group 2	54	96.43	124	2.30	66.67
Group 3	56	91.80	140	2.50	71.43
Group 4	64	98.46	170	2.66	78.12
**Partial**	240	96.77	626	2.61	70.83
Group 1	64	96.97	142	2.22	53.12
Group 2	55	98.21	139	2.53	70.91
Group 3	57	93.44	154	2.70	80.70
Group 4	64	98.46	191	2.98	79.69

Legend:
Prots = number of 248 ultra-conserved CEGs present in genome
%Completeness = percentage of 248 ultra-conserved CEGs present
Total = total number of CEGs present including putative orthologs
Average = average number of orthologs per CEG
%Ortho = percentage of detected CEGS that have more than 1 ortholog

#Listing missing proteins in each category
Category: Complete
KOG0181, KOG0261, KOG0477, KOG0969, KOG1272, KOG1374, KOG1872, KOG2303, KOG2472, KOG2707, KOG3405

Category: Partial
KOG0181, KOG0477, KOG1374, KOG1872, KOG2303, KOG2472, KOG2707, KOG3405

BUSCOs: Eudicotyledons_odb10

C: 87.8% [S:61.9%, D:25.9%], F:2.4%, M:9.8% ,n:2121
1861 Complete BUSCOs (C)
1321 Complete and single-copy BUSCOs (S)
549 Complete and duplicated BUSCOs (D)
51 Fragmented BUSCOs (F)
209 Missing BUSCOs (M)
2121 Total BUSCO groups searched

Continue on the next page

Table S2. CEGMA and BUSCO genome completeness analyses raw results. (Continuation)

(B) CEGMA and BUSCO results of *U. gibba*

Statistics of the completeness of the genome based on 248 CEGs

	#Prots	%Completeness	#Total	Average	%Ortho
**Complete**	241	97.18	404	1.68	43.98
Group 1	64	96.97	82	1.28	20.31
Group 2	53	94.64	84	1.58	41.51
Group 3	59	96.72	101	1.71	49.15
Group 4	65	100.00	137	2.11	64.62
**Partial**	243	97.98	431	1.77	47.74
Group 1	65	98.48	85	1.31	23.08
Group 2	53	94.64	91	1.72	47.17
Group 3	60	98.36	112	1.87	53.33
Group 4	65	100.00	143	2.20	67.69

Legend:
Prots = number of 248 ultra-conserved CEGs present in genome
%Completeness = percentage of 248 ultra-conserved CEGs present
Total = total number of CEGs present including putative orthologs
Average = average number of orthologs per CEG
%Ortho = percentage of detected CEGS that have more than 1 ortholog

#Listing missing proteins in each category
Category: Complete
KOG0062, KOG0209, KOG0346, KOG0434, KOG0741, KOG0894, KOG1272

Category: Partial
KOG0062, KOG0209, KOG0346, KOG0434, KOG0741

BUSCOs: Eudicotyledons_odb10

C: 88.3% [S:82.6%, D:5.7%], F:3.8%, M:7.9%, n:2121
1872 Complete BUSCOs (C)
1752 Complete and single-copy BUSCOs (S)
120 Complete and duplicated BUSCOs (D)
81 Fragmented BUSCOs (F)
168 Missing BUSCOs (M)
2121 Total BUSCO groups searched

Table S3. Mapping result of the occurrences of the eight polymorphic simple sequence repeats (SSRs) markers previously developed for Utricularia reniformis. The SSRs locus was detected using EMBOSS primersearch and BLAST tool.

	Utre04	Utre05	Utre15	Utre18	Utre19	Utre21	Utre22	Utre23
U. reniformis	2	2	1	2	none	10	1	1
U. gibba	1	1	none	none	none	none	none	none

Table S4. Association table of each *Utricularia reniformis* scaffolds to *U. gibba, Arabidopsis thaliana*, *Vitis vinifera* and *Solanum lycopersicum* scaffolds with the position of the match. They are generated by D-GENIES tool.

"Provided in a spreadsheet file"

Table S5. *Utricularia reniformis* read mapping against *U. gibba* assembled genome. Read mapping was made with bowtie2 using 'local,' '-N 1' and 'very-sensitive' parameters and CLC Genomics Workbench read mapper (length and similarity fraction set to 0.5).

	PE reads (HiScanSQ)	PE reads (MiSeq)	MP reads (HiScanSQ)
**Mapped reads in *U. gibba* (*bowtie2*)**			
- Organelles (cp and mt) reads	13,110,160 (6.7%)	2,157,794 (7%)	1,200,237 (2%)
- Genomic reads	14,983,040 (8%)	4,315,588 (12%)	3,600,712 (6%)
**Mapped reads in *U. gibba* (CLC)**			
- Organelles (cp and mt) reads	14,486,419 (7.8%)	2,206,850 (6%)	1,524,205 (2.5%)
- Genomic reads	68,920,425 (37%)	8,780,397 (24%)	13,494,238 (22.5%)

***** Single-end reads generated after the trimming process were not considered in the read mapping.

Table S6. *Utricularia reniformis* TEs repertoire identified and comparisons against the *U. gibba*.

	TEs Classification: Orders, Superfamily and Evolutionary Lineages	Number of elements		Total Occurrences		Length Occupied (bp)		% of genome	
		U.renif	*U.gibba*	*U.renif*	*U.gibba*	*U.renif*	*U.gibba*	*U.renif*	*U.gibba*
CLASS I	**SINEs**	59	14	661	79	346,565	72,514	0.11	0.07
	**LINEs**	124	9	1,659	422	1,407,662	126,974	0.46	0.13
	**LTR elements**	4,431	658	190,062	18,391	145,571,891	26,216,387	47.80	26.04
	**- LTR-LARD**	249	330	19,906	2,245	10,979,500	14,484,963	3.61	14.39
	**- LTR-TRIM**	249	62	2,633	139	3,214,503	533,891	1.06	0.53
	**- LTR: *Copia***	**1,311**	**94**	**44,241**	**7,430**	**32,473,922**	**4,392,779**	**10.66**	**4.36**
	- Ale	303	6	6,797	1,388	5,359,150	576,893	1.76	0.57
	- Alesia	2	0	11	0	8,546	0	0.01	0.00
	- Angela	3	32	25	2,356	44,620	1,338,654	0.01	1.33
	- Bianca	32	0	3,493	0	1,649,156	0	0.54	0.00
	- Ivana	186	28	4,158	1,109	2,873,891	1,164,542	0.94	1.16
	- Ikeros	41	0	850	0	1,145,829	0	0.38	0.00
	- SIRE	92	0	3,403	0	2,358,807	0	0.77	0.00
	- TAR	24	4	1,305	461	661,694	221,825	0.22	0.22
	- Tork	263	7	15,827	896	9,768,868	475,503	3.21	0.47
	- Copia/Unclassified [1]	365	17	8,372	1,220	8,603,361	615,362	2.62	0.61
	**- LTR: *Gypsy***	**1,744**	**105**	**105,322**	**5,877**	**88,902,106**	**5,028,839**	**29.19**	**4.99**
	- chromoviruses	434	28	15,778	1,908	16,152,455	1,286,233	5.30	1.28
	- CRM	20	19	612	1,145	500,459	839,088	0.16	0.83
	- Reina	21	3	112	484	123,368	199,849	0.04	0.20
	- Tekay	336	6	12,993	279	13,445,567	247,296	4.41	0.25
	- Others/Unclassified [1]	57	0	2,061	0	2,083,061	0	0.65	0.00
	- non-chromodomain	1,310	77	89,544	3,969	72,749,651	3,742,606	23.89	3.72
	- OTA/Athila	0	54	0	3,248	0	2,914,740	0.00	2.89
	- OTA/Ogre/Tat	917	1	75,146	11	63,837,472	13,420	20.96	0.01
	- OTA/Ogre/Tat/Retand	393	22	14,398	710	8,912,179	814,446	2.93	0.81
	**- LTR Unknown**	878	67	17,960	2,700	10,001,860	1,775,915	3.28	1.76
	**Others from Class I** [2]	215	4	2,994	13	2,686,704	1,754	0.88	0.01
	**Total Retroelements**	**4,829**	**685**	**195,376**	**18,905**	**150,012,822**	**26,417,629**	**49.26**	**26.24**

Continue on the next page

Table S6. *Utricularia reniformis* TEs repertoire identified and comparisons against the *U. gibba*. (Continuation)

	TEs Classification: Orders, Superfamily and Evolutionary Lineages	Number of elements		Total Occurrences		Length Occupied (bp)		% of genome	
		U.renif	*U.gibba*	*U.renif*	*U.gibba*	*U.renif*	*U.gibba*	*U.renif*	*U.gibba*
CLASS II	MITE	198	18	3,687	419	1,494,503	137,167	0.49	0.14
	Helitron	4	0	149	0	169,524	0	0.06	0.00
	TIR	1,393	187	24,749	9,476	20,375,664	4,954,221	6.69	4.92
	- *hAT*	112	13	3,531	1,021	2,507,016	632,263	0.82	0.63
	- *MuDR/Mutator*	224	25	6,829	1,410	6,105,967	953,806	2.00	0.95
	- *PIF/Harbinger*	103	24	2,533	1,559	2,045,935	772,745	0.67	0.77
	- *Others/Unclassified* [1]	954	125	2,492	1,551	2,158,080	743,536	0.71	0.74
	**Total DNA Transposons**	**1,595**	**205**	**31,077**	**11,446**	**24,197,771**	**5,834,924**	**7.95**	**5.80**

[1] Sequence classified at superfamily level and with potential several evolutionary lineages, mostly representing partial and chimeric elements sequences.

[2] Sequence classified at class level and with potential several orders, mostly representing partial and chimeric elements sequences.

Table S7. Gene Ontology (GO) self:self-genome comparative annotation raw results are showing the enrichment of each gene bin (singleton, dispersed, proximal, tandem, and segmental). They are generated with BLAST2GO tool. The GO terms were filtered for eudicotyledons clade.

"Provided in a spreadsheet file"

Table S8. KEGG enzymes self:self-genome comparative annotation raw results are showing the enrichment of each gene bin (singleton, dispersed, proximal, tandem, and segmental). They are generated with BLAST2GO tool.

"Provided in a spreadsheet file"

Table S9. Gene Ontology (GO) self:self-genome comparative annotation raw results are showing the enrichment of each gene bin (singleton, dispersed, proximal, tandem, and segmental). They are generated with GOATOOLS. The GO terms were filtered for eudicotyledons clade.

"Provided in a spreadsheet file"

Table S10. ABC transporter identified in *U. reniformis* and *U. gibba*. Total number of identified genes (Tot.) and exclusive genes (Excl. - considering the set of *U. reniformis*, *U. gibba*, *Arabidopsis thaliana*, *Vitis vinifera*, and *Lycopersicon esculentum* species) for each species.

ABC transporters	*U. renif* genes		*U. gibba* genes		Predicted and potential role in *Utricularia*
	Tot.	*Excl.*	*Tot.*	*Excl.*	
A family	6	1	6	1	Supply fatty acid substrates for triacylglycerol.
B family	36	19	14	2	Developmental processes and functions necessary for life on dry land.
C family	15	3	13	1	Developmental processes and functions necessary for life on dry land.
D family	3	-	2	-	Transport of lipid metabolic intermediates between cell compartments.
E family	6	1	1	-	Unknown.
F family	8	-	6	-	Unknown.
G family	43	17	29	11	Biotic stress - Potentially related to carnivory process.
I family	11	-	9	1	Transport of lipid metabolic intermediates between cell compartments.
Undefined	4	1	6	1	Unknown.

Table S11. Wuschel-like Homeobox TFs identified in *U. reniformis* and *U. gibba*. Total number of identified genes (Tot.) and exclusive genes (Excl. - considering the set of *U. reniformis*, *U. gibba*, *Arabidopsis thaliana*, *Vitis vinifera*, and *Lycopersicon esculentum* species) for each species.

Wuschel-like Homeobox	*U. reniformis* genes		*U. gibba* genes		Predicted and potential role in *Utricularia*
	Tot.	*Excl.*	*Tot.*	*Excl.*	
WOX1	9	2	6	3	Trap development.
WOX1-like	1	1	2	2	Trap development.
WOX2	1	-	1	-	Apical cell development.
WOX3	1	-	2	-	Vegetative and floral organs development.
WOX4	1	-	1	-	Seed development, phloem or xylem histogenesis.
WOX8	2	-	1	-	Cell division maintenance.
WOX9	3	-	3	-	Cell division maintenance.
WOX11/12	1	1	2	1	Stolon formation and growth.
WOX13	-	-	1	1	Multicellular organism development
WUSCHEL	5	-	3	2	Shoot and stolon maintenance.

Table S12. Homeobox-leucine zipper TFs identified in *U. reniformis* and *U. gibba*. Total number of identified genes (Tot.) and exclusive genes (Excl. - considering the set of *U. reniformis*, *U. gibba*, *Arabidopsis thaliana*, *Vitis vinifera*, and *Lycopersicon esculentum* species) for each species.

HD-Zip	*U. reniformis* genes		*U. gibba* genes		Predicted and potential role in *Utricularia*
	Tot.	*Excl.*	*Tot.*	*Excl.*	
ANL2	8	1	3	1	Accumulation of anthocyanins and primary root formation.
ATHB6	6	1	4	1	Abscisic acid-activated signaling pathway, response to water deprivation.
ATHB12	2	-	1	-	Response to abscisic acid and response to osmotic stress.
ATHB13	4	2	6	3	Cotyledon morphogenesis, leaf morphogenesis and stolon development.
ATHB14	7	2	3	-	Specifies adaxial leaf fates.
ATHB15	-	-	1	-	Regulation of meristem development.
ATHB40	5	-	3	-	Response to auxin.
ATHB51	9	5	9	6	Leaf morphogenesis.
ATHB52	5	-	4	-	Response to absence of light and response to blue light.
GLABRA 2	1	-	1	-	Development and maturation of trichomes and seed coat mucilage.
HAT2	1	1	-	-	Auxin-mediated morphogenesis.
HAT3	1	1	-	-	Multicellular organism development / Cellular redox status perception.
HAT4	-	-	2	-	Multicellular organism development / Cellular redox status perception.
HAT5	9	-	10	3	Leaf morphogenesis.
HAT14	2	1	2	1	Potentially involved in cellular redox status perception.
HAT22	8	5	9	6	Potentially involved in cellular redox status perception.
HDG2	-	-	1	1	Trichome formation.
HDG11	7	5	3	-	Positive regulator of drought stress tolerance, trichome formation.
HOX6-like	-	-	1	-	Unknown.
HOX11-like	5	1	2	-	Unknown.
MERISTEM L1	7	1	6	1	Plant epidermal cell differentiation and seed germination.
PDF2	1	1	-	-	Functionally redundant to ATML1 (MERISTEM L1).
REVOLUTA	1	1	3	-	Xylem and flower development, and light signaling.
ROC3-like	4	1	3	-	Development and maturation of trichomes and seed coat mucilage.
ROC5-like	2	2	1	-	Development and maturation of trichomes and seed coat mucilage.
ROC8-like	1	-	-	-	Development and maturation of trichomes and seed coat mucilage.
Unknown	2	-	2	-	Unknown.

Table S13. Agamous-like MADS-box TFs identified in *U. reniformis* and *U. gibba*. Total number of identified genes (Tot.) and exclusive genes (Excl. - considering the set of *U. reniformis*, *U. gibba*, *Arabidopsis thaliana*, *Vitis vinifera*, and *Lycopersicon esculentum* species) for each species.

MADS-box	*U. reniformis* genes		*U. gibba* genes		Predicted and potential role in *Utricularia*
	Tot.	*Excl.*	*Tot.*	*Excl.*	
AGL8	3	1	3	1	Floral development.
AGL9 (SEPALLATA3)	4	4	4	2	Inflorescence development and floral organogenesis.
AGL11	3	-	2	1	Gametophyte development and fertilization, and seed formation.
AGL15	2	-	2	-	Negative regulation of flowering.
AGL19	2	1	-	-	Promotes flowering.
AGL24	-	-	1	-	Promotes inflorescence fate in apical meristems.
AGL27	17	11	8	2	Negative regulation of flowering and prevents premature flowering.
AGL29	1	-	-	-	Unknown.
AGL61	-	-	2	1	Controls cell differentiation during female gametophyte development.
AGL62	9	6	6	3	Endosperm development.
AGL65	1	-	-	-	Regulation of pollen maturation.
AGL66	1	-	2	2	Regulation of pollen maturation.
AGL80	8	2	6	-	Controls cell differentiation during female gametophyte development.
AGL104	1	1	1	-	Regulation of pollen maturation.
FBP24-like	1	-	1	1	Unknown.
JOINTLESS	5	-	4	2	Control meristem maturation and inflorescence architecture.
SOC1	13	11	8	6	Possible role with phosphorus scavenging from trapped prey.
SVP	1	-	1	1	Inhibit floral transition in the autonomous flowering pathway.
PHERES 2-like	2	2	-	-	Unknown.
PLENA	1	1	1	1	Unknown.
Unknown	39	10	25	9	Unknown.

Table S14. Transcription factor TCP identified in *U. reniformis* and *U. gibba*. Total number of identified genes (Tot.) and exclusive genes (Excl. - considering the set of *U. reniformis*, *U. gibba*, *Arabidopsis thaliana*, *Vitis vinifera*, and *Lycopersicon esculentum* species) for each species.

TCP	*U. reniformis* genes		*U. gibba* genes		Predicted and potential role in *Utricularia*
	Tot.	*Excl.*	*Tot.*	*Excl.*	
TCP2	5	2	4	1	Morphogenesis of shoot organs.
TCP3	2	-	-	-	Morphogenesis of shoot organs.
TCP4	6	1	9	3	Morphogenesis of shoot organs.
TCP5	5	3	1	-	Morphogenesis of shoot organs.
TCP7	3	-	3	-	Regulation of circadian rhythm.
TCP8	4	-	1	-	Unknown.
TCP9	1	-	2	1	Leaf and root development.
TCP11	1	-	1	-	Regulation of circadian rhythm.
TCP12	1	-	4	1	Morphogenesis of shoot organs.
TCP13	-	-	1	1	Morphogenesis of shoot organs.
TCP14	6	1	1	-	Morphogenesis of shoot organs.
TCP15	1	-	2	-	Morphogenesis of shoot organs.
TCP19	1	-	1	-	Morphogenesis of shoot organs.
TCP20	1	-	1	-	Morphogenesis of shoot organs.
TCP21	1	1	-	-	Regulation of circadian rhythm.
TCP23	-	-	1	-	Regulation of circadian rhythm.
Unknown	11	2	8	2	Unknown.

Table S15. Spreadsheet showing the list of orthologues genes clusters. TAB:: Ugibba SHARED with Urenif. *U. gibba* and *U. reniformis* shared genes; TAB: Urenif EXCLUSIVES BASED on Ugibba. *U. reniformis* exclusive genes based on a comparison among only *U. gibba*; TAB: Ugibba EXCLUSIVES BASED on Urenif. *U. gibba* exclusive genes based on a comparison among only *U. reniformis*; TAB: Urenif CORE BASED on four genomes. *U. reniformis* core genes based on a comparison among *U. gibba, A. thaliana, V. vinifera, S. lycopersicum*; TAB: Ugibba CORE BASED on four genomes. *U. gibba* core genes based on a comparison among *U. reniformis, A. thaliana, V. vinifera, S. lycopersicum*; TAB: Urenif SINGLETONS based on four genomes. *U. reniformis* exclusive genes based on a comparison among *U. gibba, A. thaliana, V. vinifera, S. lycopersicum*; TAB: Ugibba SINGLETONS based on four genomes. *U. GIBBA* exclusive genes based on a comparison among *U. reniformis, A. thaliana, V. vinifera, S. lycopersicum*, using MCL inflation factor of 1.5.

"Provided in a spreadsheet file"

Supplementary Figures

Figure S1. K-mer spectrum analysis of *Utricularia reniformis* reads generated by the command KmerSpectrumPlot.pl from the ALLPATHS-LG. The plots presented in (**a**), (**b**) and (**c**) represent good data quality and mostly error-free and low sequencing bias. The KmerSpectrumPlot.pl indicates for an estimated genome size of 301,057,802bp and estimated genome size copy number (CN=1) of 149,356,821bp (49%) and CN > 1 = 151,700,981 (50%), with a coverage of 145x and SNP rate of ~1/106. (**a**) Highly polymorphic genome pattern depicted by kmer spectra at K=25. (**b**) Same spectra as the first but using logarithmic scales. (**c**) Cumulative fraction of all k-mers in the reads as a function of frequency, indicating that less than 10% of all the k-mers in all the reads have meager rates and are most likely associated with errors. The corrected cumulative spectrum (blue) starts a 0%, indicating an error-free data set.

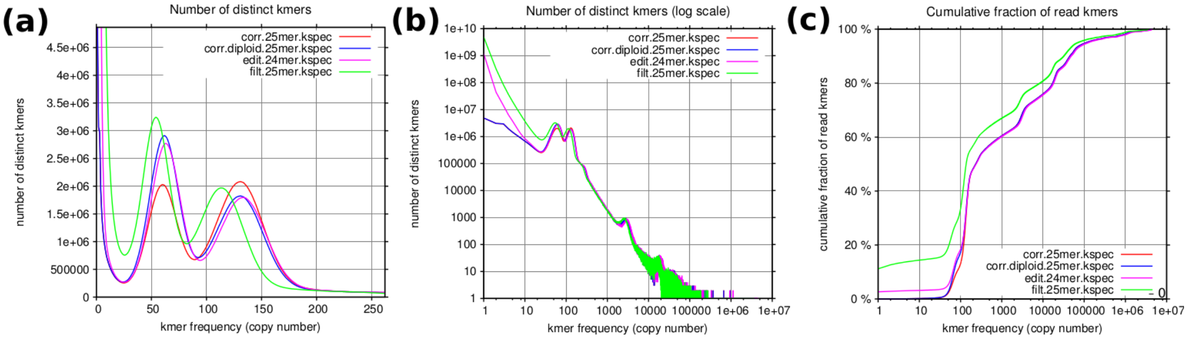

Figure S2. Microsyneny graphic overview of the occurrences of the two shared SSR markers among *Utricularia reniformis* and *U. gibba*. (**a**) Utre04 loci (**b**) Utre05 loci.

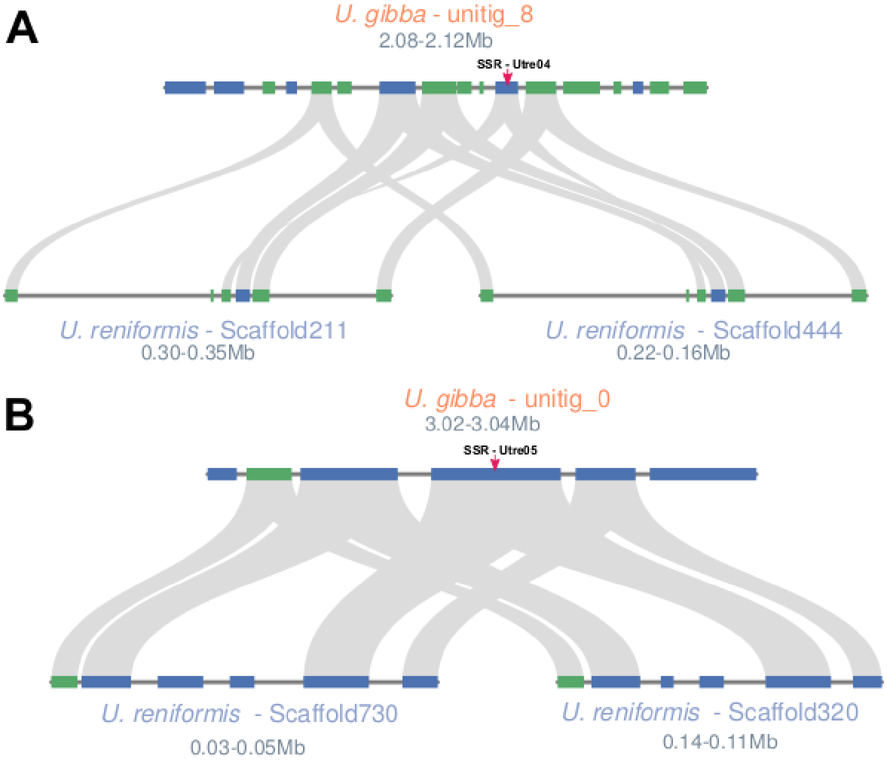

Figure S3. (**a**) *Utricularia reniformis* WGD analysis with the histogram of the rate of synonymous substitutions per synonymous site (Ks). The peak representing *U. reniformis* WGD is located between 0.31326 and 0.46243. The first peak located at 0.01492 may correspond to a large amount of unmerged and divergent heterozygous haplotypes. (**b**) Synteny pattern among *U. reniformis* and *U. gibba*.

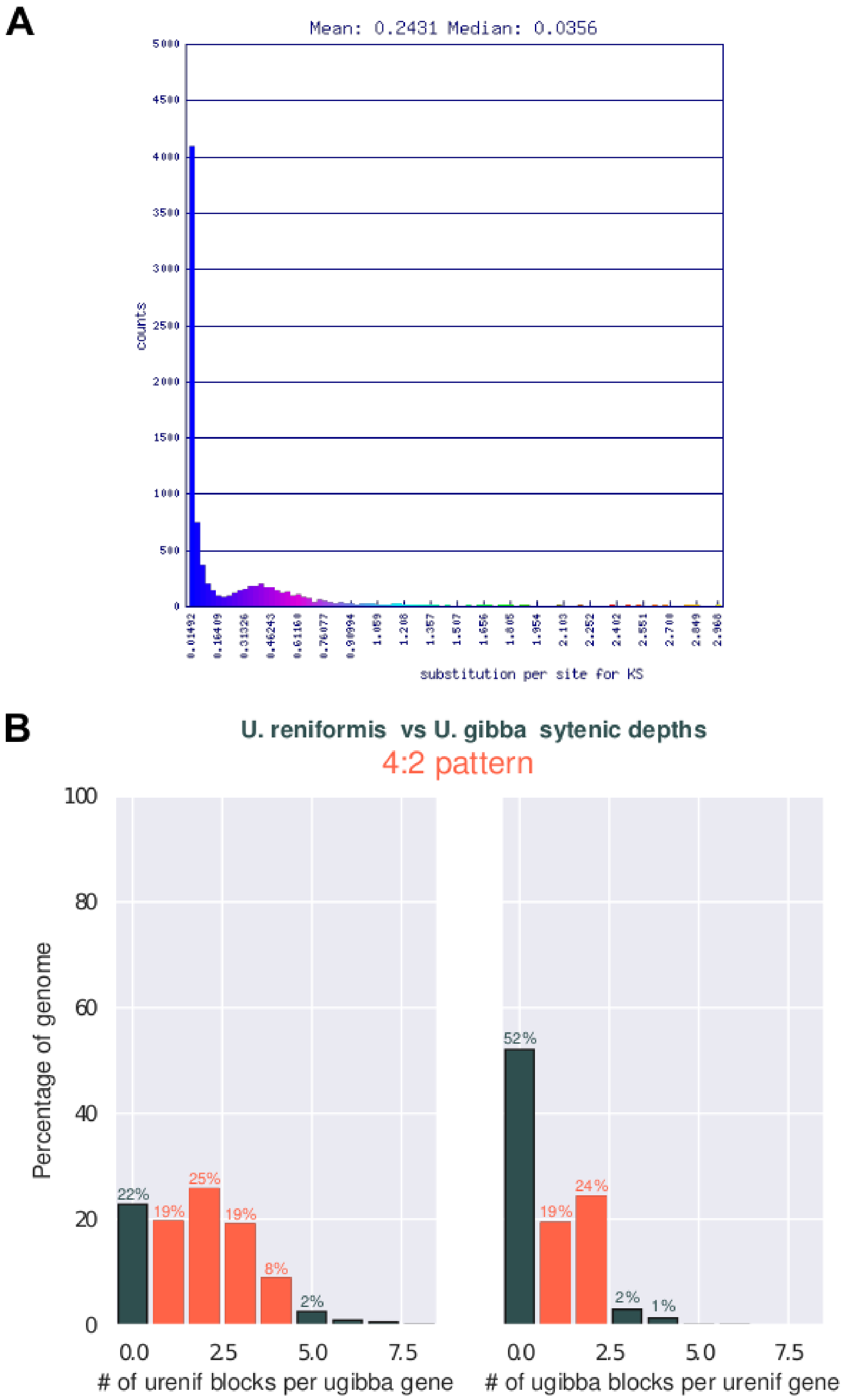

Figure S4. Bayesian inference using GTR +G +I model of ABC transport subfamilies B identified in *Utricularia reniformis* and *U. gibba*. The circles and triangles represent *U. reniformis* and *U. gibba* singletons (genes for which no orthologs could be found in any of the other species), respectively.

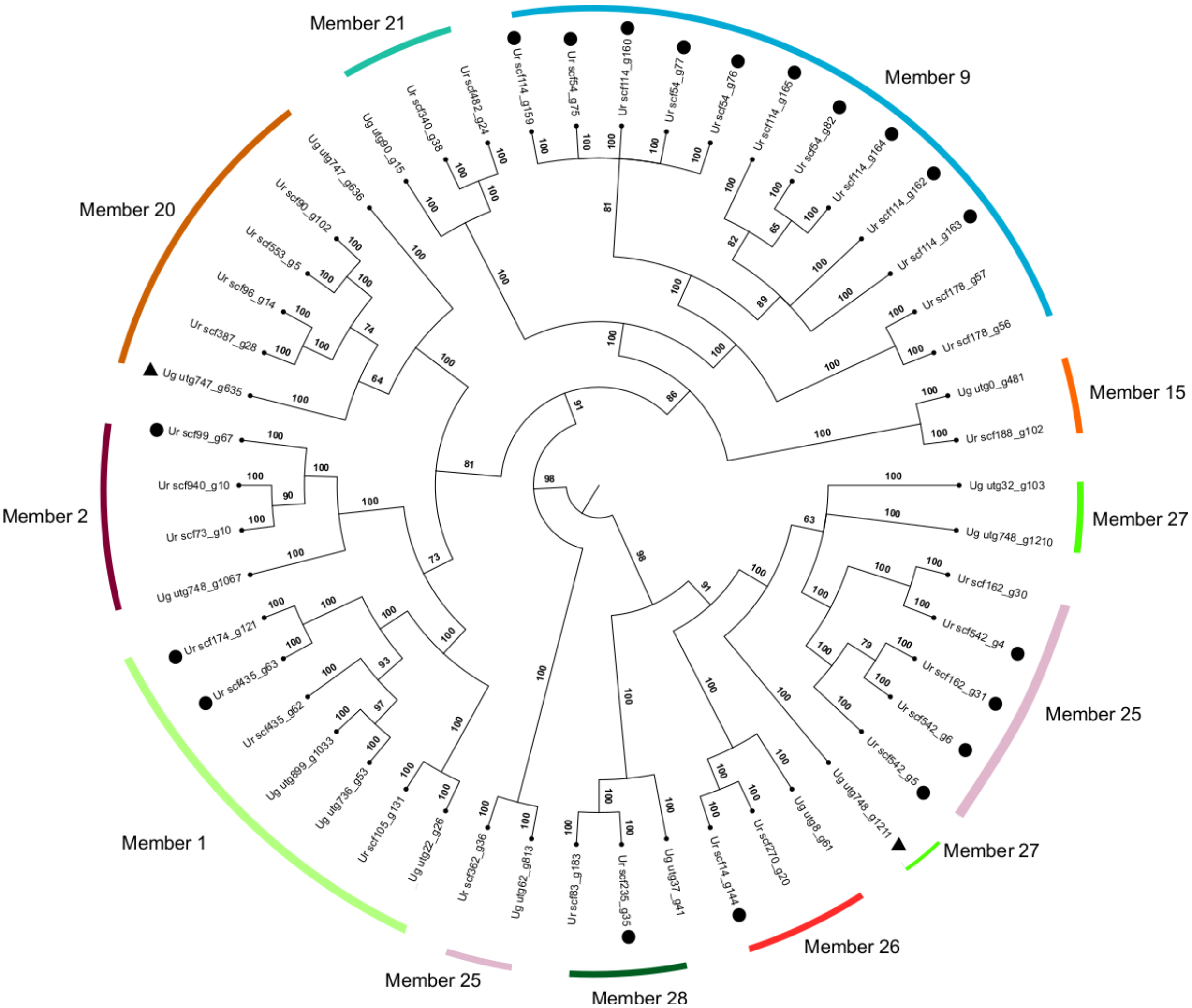

Figure S5. Bayesian inference using GTR +G +I model of ABC transport subfamily G identified in *Utricularia reniformis* and *U. gibba*. The circles and triangles represent *U. reniformis* and *U. gibba* singletons (genes for which no orthologs could be found in any of the other species), respectively.

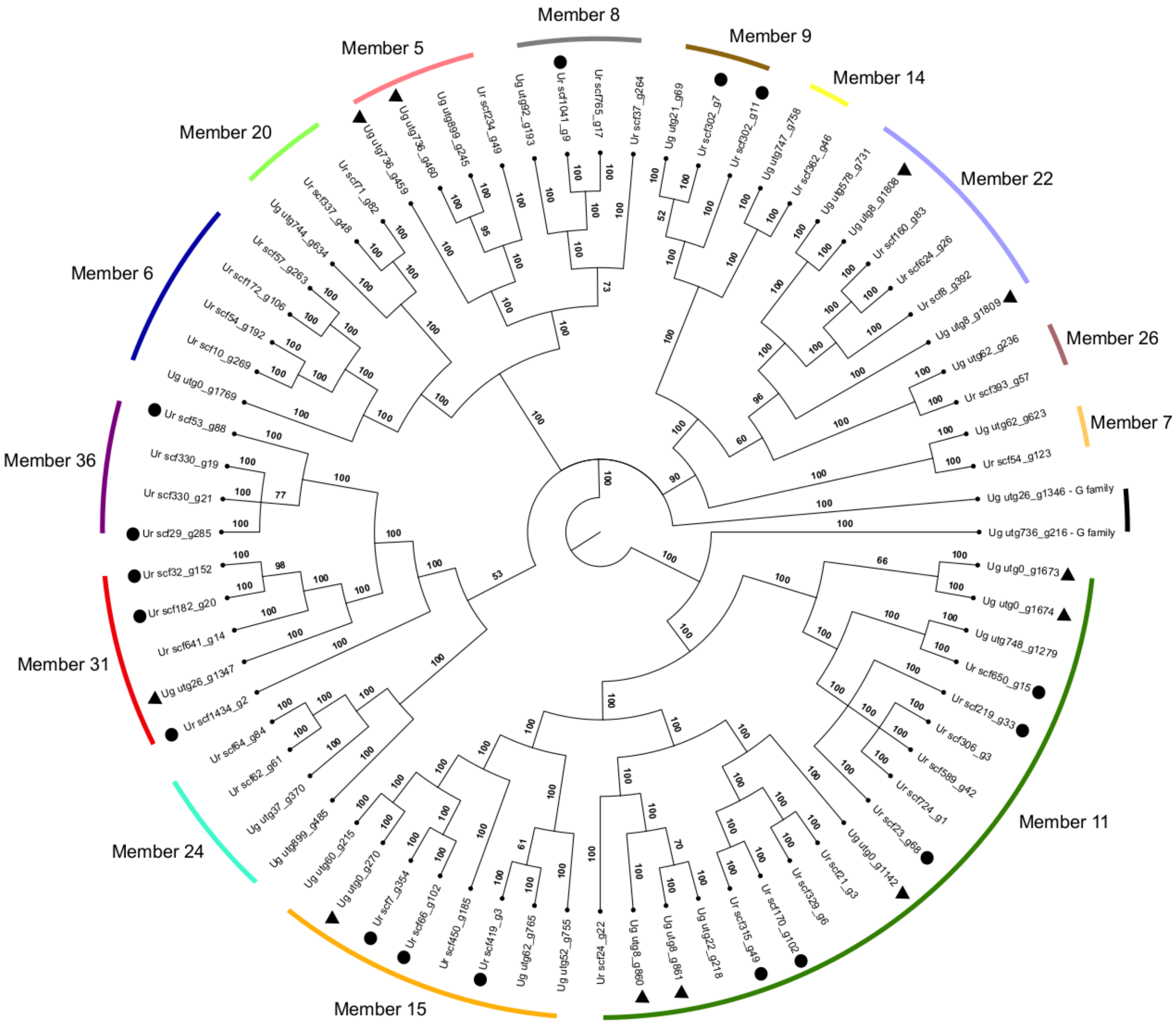

© 2019 by the authors. This is an open access supplementary information from the work "**The terrestrial carnivorous plant *Utricularia reniformis* sheds light on environmental and life-form genome plasticity**" distributed under the terms and conditions of the Creative Commons Attribution (CC BY) license (http://creativecommons.org/licenses/by/4.0/).
